# A mouse model for inherited renal fibrosis associated with endoplasmic reticulum stress

**DOI:** 10.1242/dmm.029488

**Published:** 2017-06-01

**Authors:** Sian E. Piret, Eric Olinger, Anita A. C. Reed, M. Andrew Nesbit, Tertius A. Hough, Liz Bentley, Olivier Devuyst, Roger D. Cox, Rajesh V. Thakker

**Affiliations:** 1 Academic Endocrine Unit, University of Oxford, Oxford Centre for Diabetes, Endocrinology and Metabolism, Churchill Hospital, Headington, Oxford OX3 7LJ, UK; 2 Institute of Physiology, University of Zurich, Zurich CH-8057, Switzerland; 3 School of Biomedical Sciences, University of Ulster, Coleraine BT52 1SA, UK; 4 MRC Mammalian Genetics Unit andMary Lyon Centre, MRC Harwell Institute, Harwell Science and Innovation Campus, Oxfordshire OX11 0RD, UK

**Keywords:** Kidney, Fibrosis, ER stress, Mouse model, Endoplasmic reticulum

## Abstract

Renal fibrosis is a common feature of renal failure resulting from multiple etiologies, including diabetic nephropathy, hypertension and inherited renal disorders. However, the mechanisms of renal fibrosis are incompletely understood and we therefore explored these by establishing a mouse model for a renal tubular disorder, referred to as autosomal dominant tubulointerstitial kidney disease (ADTKD) due to missense uromodulin (*UMOD*) mutations (ADTKD-*UMOD*). ADTKD-*UMOD*, which is associated with retention of mutant uromodulin in the endoplasmic reticulum (ER) of renal thick ascending limb cells, is characterized by hyperuricemia, interstitial fibrosis, inflammation and renal failure, and we used targeted homologous recombination to generate a knock-in mouse model with an ADTKD-causing missense cysteine to arginine uromodulin mutation (C125R). Heterozygous and homozygous mutant mice developed reduced uric acid excretion, renal fibrosis, immune cell infiltration and progressive renal failure, with decreased maturation and excretion of uromodulin, due to its retention in the ER. The ER stress marker 78 kDa glucose-regulated protein (GRP78) was elevated in cells expressing mutant uromodulin in heterozygous and homozygous mutant mice, and this was accompanied, both *in vivo* and *ex vivo*, by upregulation of two unfolded protein response pathways in primary thick ascending limb cells from homozygous mutant mice. However, this did not lead to an increase in apoptosis *in vivo*. Thus, we have developed a novel mouse model for renal fibrosis, which will be a valuable resource to decipher the mechanisms linking uromodulin mutations with ER stress and renal fibrosis.

## Abstract (precis)

**Summary:** A mouse model for renal fibrosis caused by uromodulin mutations reveals roles for ER stress and the unfolded protein response.

## INTRODUCTION

Renal fibrosis is an integral factor in progression to end-stage renal failure (ESRF), regardless of the primary etiology, which may include diabetic nephropathy, hypertension or monogenic disorders (Eddy, 2014). However, mechanisms of renal fibrosis are incompletely understood, and there are currently no therapies to reverse or slow down the progression of renal fibrosis (Eddy, 2014). Autosomal dominant tubulointerstitial kidney disease (ADTKD), a monogenic cause of renal fibrosis, is characterized by urine concentrating defects, interstitial nephropathy with immune cell infiltration and glomerulosclerosis, and progressive loss of renal function leading to ESRF (Eckardt et al., 2015). Mutations in four genes have been identified to cause ADTKD, with the most common being in the *UMOD* gene, encoding uromodulin (Eckardt et al., 2015; Hart et al., 2002; Turner et al., 2003), referred to as ADTKD-*UMOD*. ADTKD-*UMOD* was previously referred to as familial juvenile hyperuricemic nephropathy type 1 (FJHN1; OMIM #162000), medullary cystic kidney disease type 2 (MCKD2; OMIM #603860) and glomerulocystic kidney disease (GCKD; OMIM #609886) (Eckardt et al., 2015; Rampoldi et al., 2003). In addition to the general clinical characteristics of ADTKD, patients with ADTKD-*UMOD* present with elevated serum urate concentrations due to low fractional excretion of uric acid (FEUA).

Uromodulin, a 640 amino acid glycosylphosphatidylinositol (GPI) anchored protein, is only expressed in the thick ascending limb (TAL) of the loop of Henle (Rampoldi et al., 2011). Uromodulin is synthesized within the endoplasmic reticulum (ER), trafficked to the apical plasma membrane, cleaved into the tubule and excreted in the urine, where it is the most abundant protein (Rampoldi et al., 2011). Uromodulin likely contains: four epidermal growth factor (EGF)-like domains, of which the second and third are calcium-binding (cb) EGF domains; a cysteine-rich region, which includes a domain of eight cysteines; and a zona pellucida (ZP) domain (Bokhove et al., 2016) (Fig. S1). Several functions have been postulated for uromodulin, including: regulating sodium transport in the TAL by modulating the activity of the apical transporters NKCC2 and ROMK (renal outer medullary potassium channel) (Trudu et al., 2013); inhibiting renal stone formation; preventing bacterial colonization; and modulating immune responses (Bates et al., 2004; Darisipudi et al., 2012; Mattey and Naftalin, 1992; Mo et al., 2004a,b, 2007; Raffi et al., 2009, 2005; Säemann et al., 2005a,b).

Over 90% of the >55 reported ADTKD-*UMOD* mutations are missense, suggesting a dominant negative disease mechanism, and >60% cause loss or gain of a cysteine residue (Moskowitz et al., 2013), implicating a role for protein misfolding in ADTKD-*UMOD*. Most mutations are clustered in the EGF domains and cysteine-rich region, which are likely involved in formation of most of the 24 disulfide bridges in uromodulin (Rampoldi et al., 2011), and uromodulin missense mutations cause altered intracellular trafficking, with retention of immature, core-glycosylated uromodulin in the ER, resulting in reduced or absent expression at the plasma membrane with little or no secretion (Bernascone et al., 2006; Choi et al., 2005; Dahan et al., 2003; Jennings et al., 2007; Rampoldi et al., 2003; Vylet'al et al., 2006; Williams et al., 2009). However, the role of uromodulin mutations in causing renal interstitial fibrosis, glomerulosclerosis and immune cell infiltration is not fully understood.

ER accumulation of mutant proteins can cause ER stress, which activates the unfolded protein response (UPR), which may be adaptive or apoptotic in different tissues (Tsang et al., 2010), and consists of three pathways: the activating transcription factor-6α (ATF6α), inositol-requiring enzyme-1 (IRE1) and RNA-activated protein kinase-like ER kinase (PERK) pathways. These act to upregulate chaperones and ER-associated degradation (ERAD) components, and reduce global protein translation to decrease the ER protein load. Under prolonged and unresolved ER stress, cells may also undergo apoptosis (Tsang et al., 2010). The ER stress-induced chaperone 78 kDa glucose-regulated protein [GRP78; also known as binding immunoglobulin protein (BiP)] is upregulated in renal biopsies from patients harboring mutations in *UMOD* (Adam et al., 2012); however, mechanisms of ER stress and the UPR pathways have not been studied further in ADTKD.

Two *Umod* mouse knock-out models have been developed, which have revealed roles for uromodulin in: defense against bladder and urinary tract bacterial infections (Bates et al., 2004; Mo et al., 2004b; Raffi et al., 2009, 2005); prevention of renal stone formation (Liu et al., 2010; Mo et al., 2004a, 2007); and water impermeability and solute handling in the TAL (Bachmann et al., 2005; Mutig et al., 2011; Renigunta et al., 2011; Wolf et al., 2013). However, *Umod* knock-out mice up to 3 years of age do not develop any of the histological features or renal failure characteristic of ADTKD (Raffi et al., 2006). A transgenic mouse model carrying a human *UMOD* transgene containing the ADTKD-*UMOD*-causing mutation Cys148Trp (C148W) in addition to the two mouse *Umod* alleles was not reported to have renal impairment or abnormal renal histology (Takiue et al., 2008a,b) (Table S1). However, a second transgenic mouse expressing mouse *Umod* with the C148W mutation developed several features of ADTKD-*UMOD*, including mild renal failure, urinary concentrating defects, and interstitial inflammation and fibrosis, but decreased uric acid excretion was not reported (Bernascone et al., 2010) (Table S1). Thus, two similar transgenic models demonstrated two different phenotypes and, since in both models uromodulin was overexpressed compared to in non-transgenic mice, these models require cautious interpretation. An *N*-ethyl-*N*-nitrosourea (ENU)-generated uromodulin mutant [Ala227Thr (A227T)] mouse has been described to develop azotemia, impaired urine concentrating ability and reduced excretion of uric acid, but not renal fibrosis (Kemter et al., 2009), whilst a second ENU mutant, Cys93Phe (C93F), developed renal failure, urinary concentrating defects, interstitial fibrosis, inflammation and tubular defects (Kemter et al., 2013) (Table S1). However, neither A227T nor C93F are known disease-causing mutations in humans.

In order to study mechanisms of renal fibrosis, ADTKD-*UMOD* and ER stress due to uromodulin mutations relevant to ADTKD-*UMOD* in humans, we generated a targeted mouse knock-in model by homologous recombination in mouse embryonic stem (ES) cells, to produce mice carrying a known disease-causing mutation, Cys125Arg (C125R), within the mouse *Umod* gene. Cysteine 125 (C125) is located within the cbEGF3 domain of uromodulin, and mutation of this residue likely disrupts formation of the Ca^2+^-binding pocket which is formed by a set pattern of disulfide bonding within this domain (Turner et al., 2003) (Fig. S1). C125 in mouse uromodulin is the equivalent of C126 in human uromodulin, and mutation of this residue in humans to an arginine (C126R) has been reported to cause ADTKD-*UMOD* (Lhotta et al., 1998; Turner et al., 2003). Furthermore, C126R uromodulin is retained in the ER of transfected cells (Williams et al., 2009), which is typical of several uromodulin mutations that have been characterized *in vitro* (Bernascone et al., 2006; Choi et al., 2005; Jennings et al., 2007; Rampoldi et al., 2003; Vylet'al et al., 2006; Williams et al., 2009). This mutation was therefore selected as being representative of the majority of disease-causing uromodulin mutations.

## RESULTS

### Introduction of C125R mutation into the mouse germline

**Fig. 1.**
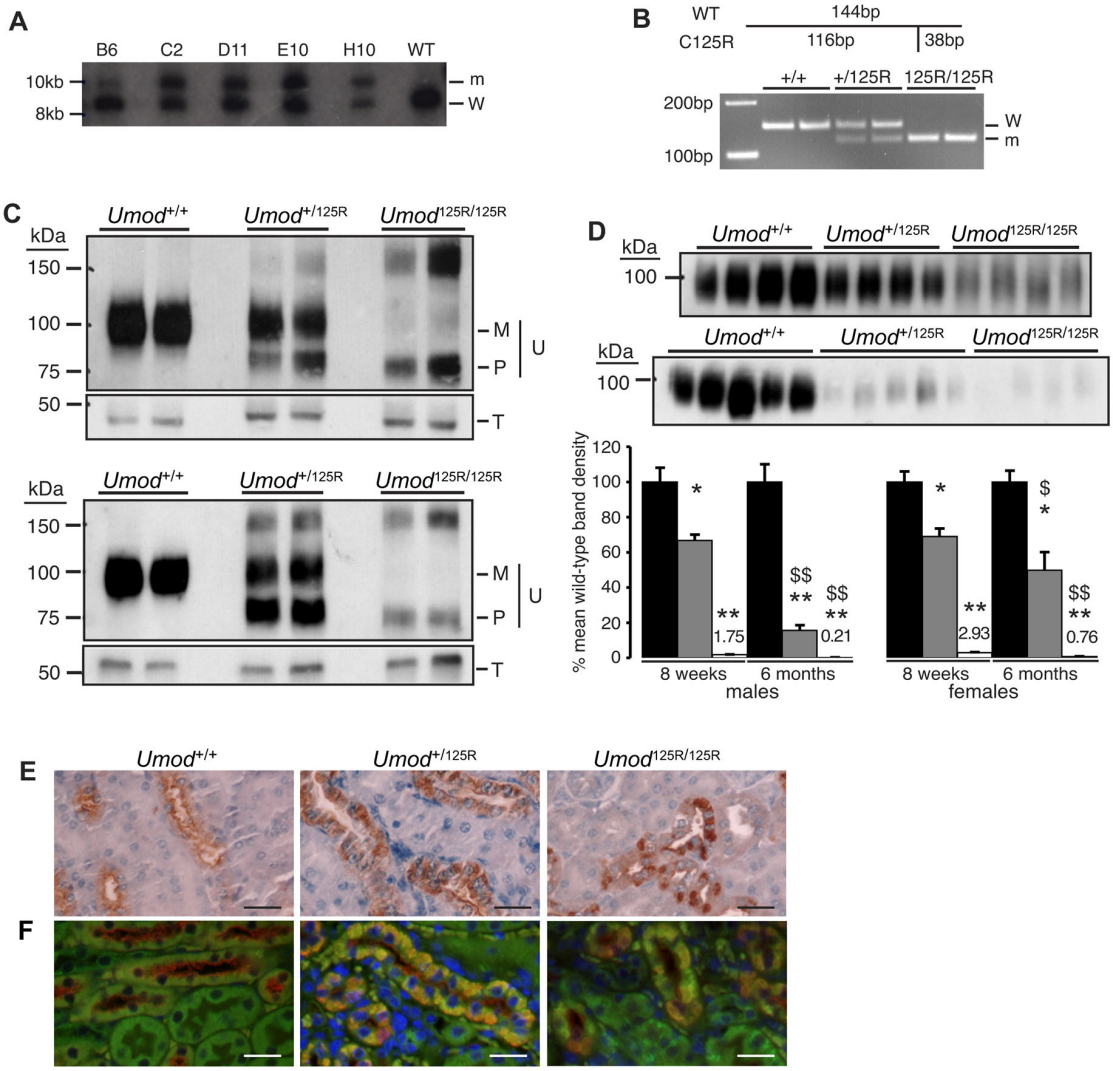
**Uromodulin maturation, excretion and trafficking.** (A) Homologous recombination in ES cell clones assessed by Southern blot analysis: DNA from 5 clones (B6, C2, D11, E10, H10) and a wild-type (WT) control underwent *Spe*I digestion, generating products of ∼8.3 kb [wild-type (W) allele] and ∼9.6 kb [mutant (m) allele]. The probe for detection spanned *Umod* exon 6. (B) Genotyping of mice: the C125R mutation introduced an additional *Bso*BI site, which was used to digest PCR products of part of exon 3, leaving the wild-type (W) 144 bp product uncut, and cleaving the mutant product into 116 bp (m) and 38 bp (not shown) fragments. Digests from two each of wild-type (+/+), heterozygous (+/125R) and homozygous mutant (125R/125R) mice are shown. (C) Western blot analysis of whole kidney lysates from *Umod*^+/+^, *Umod*^+/125R^ and *Umod*^125R/125R^ male mice aged 8 weeks (top) and 6 months (bottom). *Umod*^+/+^ mice had only the mature form (M) of uromodulin (U), but *Umod*^+/125R^ and *Umod*^125R/125R^ mice had varying amounts of the precursor form (P), and high molecular mass uromodulin that may be dimers of the precursor. Tubulin (T) was used as a control. For technical reasons, in the lower panel of C, the same lysates were blotted for uromodulin and tubulin on two different gels. (D) Representative western blots of urine from males aged 8 weeks (upper blot) and 6 months (lower blot), with densitometric analysis. *Umod*^+/+^ and *Umod*^+/125R^ samples diluted 1/20; *Umod*^125R/125R^ samples undiluted. Densitometric measurements are plotted relative to mean of *Umod*^+/+^ densities (set to 100%). *Umod*^+/+^: black bars; *Umod*^+/125R^: gray bars; *Umod*^125R/125R^: white bars. Values shown as mean+s.e.m.; *n*=9 for 8-week-old females, *n*=8 for 8-week-old males, *n*=7 for 6-month-old *Umod*^+/+^ females, *n*=8 for 6-month-old *Umod*^+/125R^ and *Umod*^125R/125R^ females, *n*=10 for 6-month-old *Umod*^+/+^ males, *n*=12 for 6-month-old *Umod*^+/125R^ males, *n*=6 for 6-month-old *Umod*^125R/125R^ males. *P<0.001, **P<0.00001 versus *Umod*^+/+^ littermates of the same age and sex. ^$^*P*<0.05, ^$$^*P*<0.00001 versus 8-week-old mice of the same sex and genotype (Student's unpaired 1-tailed *t*-test). (E) Immunohistochemical analysis of uromodulin showing apical expression in *Umod*^+/+^ mice, partial intracellular retention in *Umod*^+/125R^ mice, and dense intracellular deposits in *Umod*^125R/125R^ mice. Nuclei counterstained with hematoxylin; scale bars: 25 µm. (F) Co-immunofluorescent labeling for uromodulin (red) and calnexin (green), showing lack of colocalization in *Umod*^+/+^ kidneys, and partial and almost total colocalization in *Umod*^+/125R^ and *Umod*^125R/125R^ kidneys, respectively. Nuclei counterstained with DAPI; scale bars: 25 µm.

A targeting vector containing 7.6 kb of the mouse *Umod* gene, with the C125R mutation in exon 3, a thymidine kinase (TK) cassette at the 5′ end and a neomycin resistance (Neo^R^) cassette in intron 2, was used to introduce the C125R mutation into the mouse genome by homologous recombination in ES cells (Fig. S1). Of 272 ES cell clones that were selection resistant, 5 clones, termed B6, C2, D11, E10 and H10, were shown to have undergone homologous recombination at the 5′ and 3′ ends of the targeting construct by PCR/restriction assay (data not shown) and Southern blot analysis (Fig. 1A). Blastocyst injection of clone C2 resulted in the birth of chimeras with a high degree of chimerism (>∼90%). Male chimeras were bred with C57BL/6J females and the mutation was shown to enter the germline by genotyping of mice using PCR and *Bso*BI restriction digest (Fig. 1B). Germline mutant mice were bred with β-actin-Cre mice to excise the Neo^R^ cassette. Heterozygous (*Umod*^+/125R^) and homozygous (*Umod*^125R/125R^) mutant mice were viable and fertile, with appearances and body weights (Table 1) similar to wild-type (*Umod*^+/+^) mice.

**Table 1.**
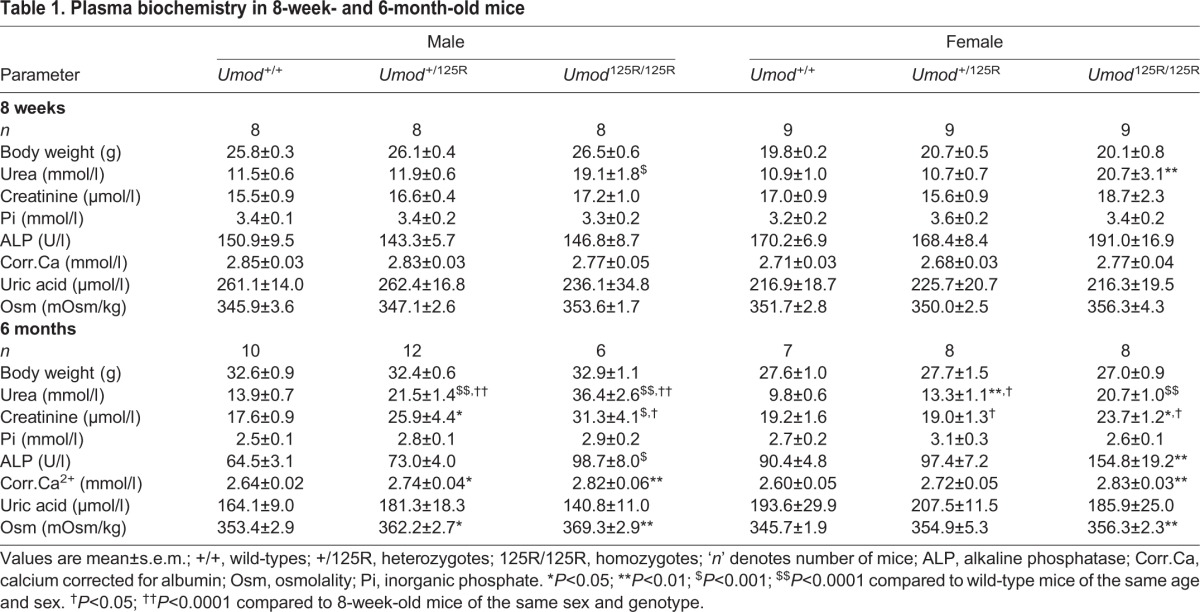
**Plasma biochemistry in 8-week- and 6-month-old mice**

### Plasma biochemistry in 8-week- and 6-month-old *Umod*^+/+^, *Umod*^+/125R^ and *Umod*^125R/125R^ mice

Male (*P*<0.001) and female (*P*<0.01) *Umod*^125R/125R^ mice had plasma urea levels ∼2-fold greater than those of *Umod*^+/+^ and *Umod*^+/125R^ littermates at 8 weeks of age (Table 1). By 6 months of age, plasma urea levels were increased ∼1.5-fold and ∼2.5-fold in *Umod*^+/125R^ (male *P*<0.0001; female *P*<0.01) and *Umod*^125R/125R^ (male *P*<0.0001; female *P*<0.0001) mice, respectively, compared to *Umod*^+/+^ littermates (Table 1). Plasma urea increased between 8-week- and 6-month-old *Umod*^+/125R^ mice by ∼1.8-fold (*P*<0.0001) in males and ∼1.2-fold (*P*<0.05) in females, demonstrating progressive renal failure (Table 1). Plasma creatinine was also significantly raised, by ∼1.5-fold and ∼1.8-fold, in 6-month-old *Umod*^+/125R^ (*P*<0.05) and *Umod*^125R/125R^ (*P*<0.001) males, respectively, and by ∼1.2-fold in *Umod*^125R/125R^ females (*P*<0.05) compared to age and sex matched *Umod*^+/+^ littermates (Table 1). Male and female 6-month-old *Umod*^125R/125R^ mice also had significantly raised plasma corrected calcium concentrations (*P*<0.01) compared to *Umod*^+/+^ littermates, which, in conjunction with a significantly raised plasma alkaline phosphatase (ALP) activity (males *P*<0.001; females *P*<0.01), is consistent with the likely onset of tertiary hyperparathyroidism (Table 1). However, this hypercalcemia in association with raised plasma ALP activity in the *Umod*^125R/125R^ mice, which has also previously been reported in two ENU-induced mutant mouse models with A227T and C93F *Umod* mutations (Kemter et al., 2009, 2013) (Table S1), has not been observed to occur in ADTKD-*UMOD* patients who develop renal failure. A plausible explanation for this occurrence of hypercalcemia, likely due to tertiary hyperparathyroidism, in *Umod* mutant mice but not ADTKD-*UMOD* patients is that the earlier detection of renal failure and associated secondary hyperparathyroidism, with appropriate treatment using vitamin D analogs, phosphate binders and calcimimetics, will prevent the progression to tertiary hyperparathyroidism in patients. Plasma uric acid levels were not increased in *Umod*^+/125R^ or *Umod*^125R/125R^ mice at 8 weeks or 6 months, likely due to expression of the hepatic enzyme uricase in mice (Wu et al., 1992). Plasma sodium and potassium concentrations were similar in mutant and wild-type mice (data not shown).

### Urine biochemistry in 8-week- and 6-month-old *Umod*^+/+^, *Umod*^+/125R^ and *Umod*^125R/125R^ mice

Male and female *Umod*^125R/125R^ mice excreted ∼2–4-fold increased volumes of urine (corrected for body weight) (*P*<0.0001), and this urine was more dilute with ∼2–4-fold decreased urine osmolality (8-week-old males and 6-month-old males and females *P*<0.0001; 8-week-old females *P*<0.05) compared to *Umod*^+/+^ littermates (Table 2). In addition, male *Umod*^+/125R^ mice aged 8 weeks (*P*<0.05) and male and female *Umod*^+/125R^ mice aged 6 months (*P*<0.05) had reduced urine osmolality, and 6-month-old male *Umod*^+/125R^ mice also excreted 1.5-times more urine (*P*<0.05) compared to *Umod*^+/+^ littermates (Table 2). In 6-month-old *Umod*^125R/125R^ males and females (*P*<0.01), and *Umod*^+/125R^ males (*P*<0.05), this large excretion of dilute urine caused plasma osmolality to rise and, in other mutant mice, plasma osmolalities were inappropriately normal (Table 1). Thus, *Umod*^+/125R^ and *Umod*^125R/125R^ mice have a urine concentrating defect, which is similar to that reported in ADTKD-*UMOD* patients (Rampoldi et al., 2003; Scolari et al., 2004).

**Table 2.**
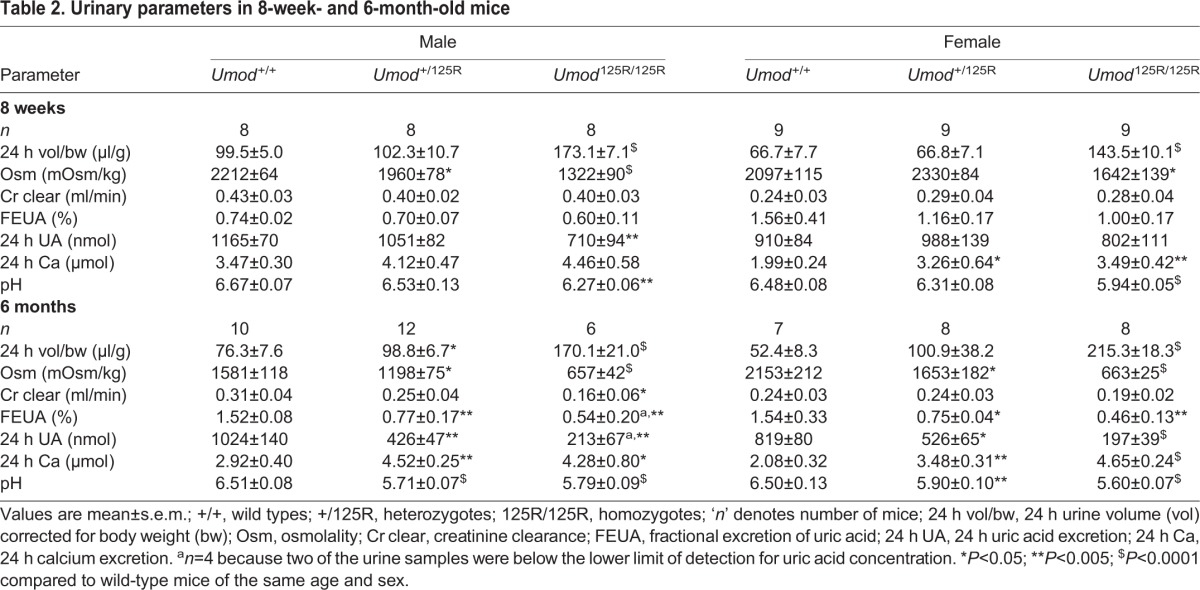
**Urinary parameters in 8-week- and 6-month-old mice**

Twenty-four hour uric acid excretion was decreased in 6-month-old mice by up to ∼2.4-fold in *Umod*^+/125R^ mice (males *P*<0.005; females *P*<0.05), and up to ∼4.8-fold in *Umod*^125R/125R^ mice (males *P*<0.005; females *P*<0.0001) compared to *Umod*^+/+^ littermates (Table 2). In 8-week-old *Umod*^125R/125R^ males, 24 h uric acid excretion was also decreased by ∼1.6-fold compared to *Umod*^+/+^ male littermates (*P*<0.005) (Table 2). This was not due to the dilute urine produced by these mice, since the 24 h excretion takes urine volume into account. In 6-month-old mice, *Umod*^+/125R^ and *Umod*^125R/125R^ male and female mice had a significantly (*P*<0.05 to *P*<0.005) reduced FEUA, compared to *Umod*^+/+^ littermates (Table 2). This specific reduction in uric acid excretion in *Umod*^+/125R^ and *Umod*^125R/125R^ mice is analogous to the human ADTKD-*UMOD* phenotype, in which >90% of patients have a decreased FEUA (Williams et al., 2009).

Urinary calcium excretion was elevated by ∼1.6-fold (*P*<0.05) and ∼1.8-fold (*P*<0.005) in 8-week-old female *Umod*^+/125R^ and *Umod*^125R/125R^ mice, respectively, and elevated by ∼1.5-fold in *Umod*^+/125R^ (*P*<0.005) and *Umod*^125R/125R^ (*P*<0.05) 6-month-old males, and ∼1.6-fold (*P*<0.005) and ∼1.8-fold (*P*<0.0001) in *Umod*^+/125R^ and *Umod*^125R/125R^ 6-month-old females, respectively, when compared to *Umod*^+/+^ littermates (Table 2). This hypercalciuria of *Umod* mutant mice, which has not been described in human ADTKD-*UMOD* patients, was associated with urinary acidification. Thus, urinary pH was significantly reduced in 8-week-old *Umod*^125R/125R^ mice, corresponding to ∼1.5-fold (*P*<0.005) and ∼2.5-fold (*P*<0.0001) increases in urinary H^+^ ion concentrations in males and females, respectively, compared to *Umod*^+/+^ littermates (Table 2). Six-month-old *Umod*^+/125R^ and *Umod*^125R/125R^ mice also had a decreased urinary pH corresponding to up to ∼5.3-fold (*P*<0.0001) and ∼7-fold (*P*<0.005) increases in urinary H^+^ ion concentrations in males and females, respectively, compared to *Umod*^+/+^ littermates (Table 2). This urinary acidification helps to prevent renal stone formation, and is a likely renal response to hypercalciuria (Renkema et al., 2009).

### Uromodulin maturation, excretion and trafficking in 8-week- and 6-month-old *Umod*^+/125R^ and *Umod*^125R/125R^ mice

The C126R uromodulin mutation results in defective maturation, trafficking and secretion of uromodulin *in vitro* (Williams et al., 2009), and we therefore carried out western blot analysis of kidney lysates and urine, and immunohistochemistry, to study this in *Umod* mutant mice. Kidney lysates from *Umod*^+/+^ mice showed the presence of uromodulin at ∼100 kDa as previously described (Bates et al., 2004), corresponding to mature, fully glycosylated uromodulin (Fig. 1C). Kidney lysates from *Umod*^+/125R^ mice at 8 weeks and 6 months of age had mature uromodulin and a lower molecular mass form of uromodulin, likely the ER-resident precursor, which has core glycosylations only (Williams et al., 2009). Kidney lysates from *Umod*^125R/125R^ mice almost entirely lacked mature uromodulin, with a predominance of the precursor form of uromodulin. Higher molecular mass bands were detected in kidney lysates from *Umod*^+/125R^ and *Umod*^125R/125R^ mice at 8 weeks and 6 months, at approximately double the molecular mass of the precursor uromodulin; these may represent dimers of precursor uromodulin, which would likely be retained in the ER (Fig. 1C).

Urinary uromodulin excretion was studied by western blot and densitometric analysis (Fig. 1D). Uromodulin is the most abundant protein in the urine (Scolari et al., 2015); therefore, to enable accurate western blot analysis, samples from *Umod*^+/+^ and *Umod*^+/125R^ mice were diluted 20-fold, but samples from *Umod*^125R/125R^ mice were not diluted. Sample loading was standardized for equal creatinine concentration, to correct for the more dilute urine in mutant mice. Eight-week-old male and female *Umod*^+/125R^ mice had a significantly reduced uromodulin excretion of 66.8±3.3% and 69.1±4.5%, respectively, compared to *Umod*^+/+^ littermates (*P*<0.001) (Fig. 1D). This preceded the onset of renal failure, as measured by an increase in plasma urea (Table 1). Furthermore, this decrease in uromodulin excretion was progressive, since both *Umod*^+/125R^ and *Umod*^125R/125R^ mice at 6 months old excreted significantly less uromodulin than mice of the same sex and genotype aged 8 weeks (Fig. 1D). For example, uromodulin excretion in male *Umod*^+/125R^ mice decreased from 49.8±10.3% at 8 weeks of age to 15.7±2.9% at 6 months of age when compared to *Umod*^+/+^ littermates (*P*<0.00001). All of the excreted uromodulin was the mature form of uromodulin at ∼100 kDa, with no detectable excretion of the precursor form of uromodulin.

Immunohistochemical analysis of uromodulin expression in 8-week- and 6-month-old *Umod*^+/+^, *Umod*^+/125R^ and *Umod*^125R/125R^ kidneys showed the expected uromodulin trafficking defect in mutant kidneys (Fig. 1E). In *Umod*^+/+^ kidneys, uromodulin expression had a predominantly apical pattern, with low levels of diffuse intracellular uromodulin also present as previously described (Kemter et al., 2009), and uromodulin was observed in the tubular lumen. In *Umod*^+/125R^ and *Umod*^125R/125R^ kidneys, apical expression was decreased, and dense intracellular expression was observed, such that, in *Umod*^+/125R^ kidneys, uromodulin was partly apical and partly intracellular, and, in *Umod*^125R/125R^ kidneys, uromodulin was almost exclusively located in dense intracellular deposits. Dual-labeling for uromodulin and the ER marker calnexin showed that the intracellular uromodulin in *Umod*^+/125R^ and *Umod*^125R/125R^ kidneys was colocalized with the ER (Fig. 1F), as previously demonstrated both *in vitro* (Williams et al., 2009) and in ADTKD-*UMOD* patients (Bernascone et al., 2010), and consistent with the presence of the lower molecular mass precursor detected by western blotting (Fig. 1C). In *Umod*^+/125R^ mouse kidneys, uromodulin colocalized with calnexin and was present at the apical membrane.

### Renal fibrosis and immune cell infiltration in 8-week- and 6-month-old *Umod*^+/125R^ and *Umod*^125R/125R^ mice

**Fig. 2.**
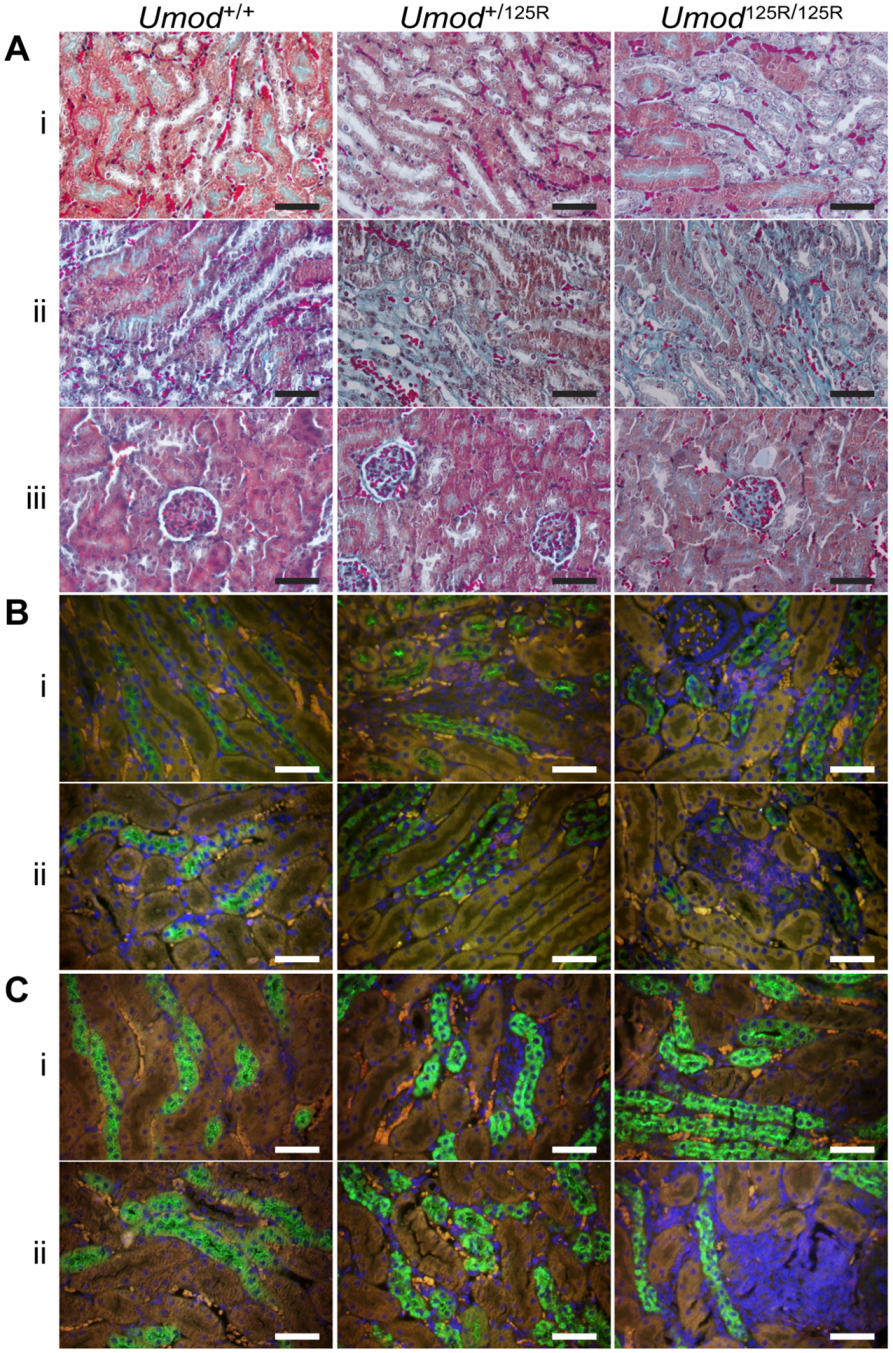
**Fibrosis and inflammation in *Umod*^+/+^, *Umod*^+/125R^ and *Umod*^125R/125R^ kidneys.** (A) Masson's trichrome stain in the corticomedullary region at (i) 8 weeks and (ii) 6 months, and (iii) in the cortex at 6 months, showing fibrous material (blue). (B) Co-immunofluorescent labeling for uromodulin (green) and the T-cell marker CD3 (red) at (i) 8 weeks and (ii) 6 months. Nuclei counterstained with DAPI. (C) Co-immunofluorescent labeling for uromodulin (green) and the macrophage marker F4/80 (red) at (i) 8 weeks and (ii) 6 months. Nuclei are counterstained with DAPI. All scale bars: 50 µm.

Masson's trichrome staining, to study renal damage and fibrosis, of kidney sections from 8-week- and 6-month-old mice revealed that, at 8 weeks, kidneys of *Umod*^+/125R^ mice did not have any abnormalities, but that kidneys of *Umod*^125R/125R^ mice had thickened basement membranes and some intracellular fibrous deposits, similar to those reported in a renal biopsy from a patient with a *UMOD* mutation (Nasr et al., 2008). Glomeruli appeared normal in both *Umod*^+/125R^ and *Umod*^125R/125R^ mouse kidneys at 8 weeks of age (Fig. 2A). At 6 months of age, *Umod*^+/125R^ mouse kidneys had mild interstitial fibrosis, and *Umod*^125R/125R^ mouse kidneys had large areas of fibrosis, with some renal tubules appearing to become detached from the basement membrane. Furthermore, some glomeruli in *Umod*^125R/125R^ mouse kidneys also had increased fibrotic material, consistent with glomerulosclerosis (Fig. 2A). No cysts were observed in *Umod*^+/125R^ or *Umod*^125R/125R^ mouse kidneys at 8 weeks or 6 months of age.

During analysis of uromodulin-labeled and trichrome-stained kidney sections, interstitial cell infiltrates were noted. To determine the nature of these infiltrates, kidney sections were labeled for T-cell (CD3) and macrophage (F4/80) markers. *Umod*^+/125R^ mouse kidneys at 8 weeks of age had no detectable immune cell infiltration; however, *Umod*^125R/125R^ mouse kidneys showed small areas of interstitial infiltrates, consisting of T-cells and macrophages (Fig. 2B,C). At 6 months of age, *Umod*^+/125R^ mouse kidneys showed small areas of interstitial T-cells and macrophages, but *Umod*^125R/125R^ mouse kidneys had multiple, large areas of interstitial infiltrates, consisting of both T-cells and macrophages (Fig. 2B,C). These infiltrates occurred mainly in the medullary region, and often in close proximity to TAL cells expressing mutant uromodulin.

### Upregulation of ER stress and UPR pathways in mutant TALs

**Fig. 3.**
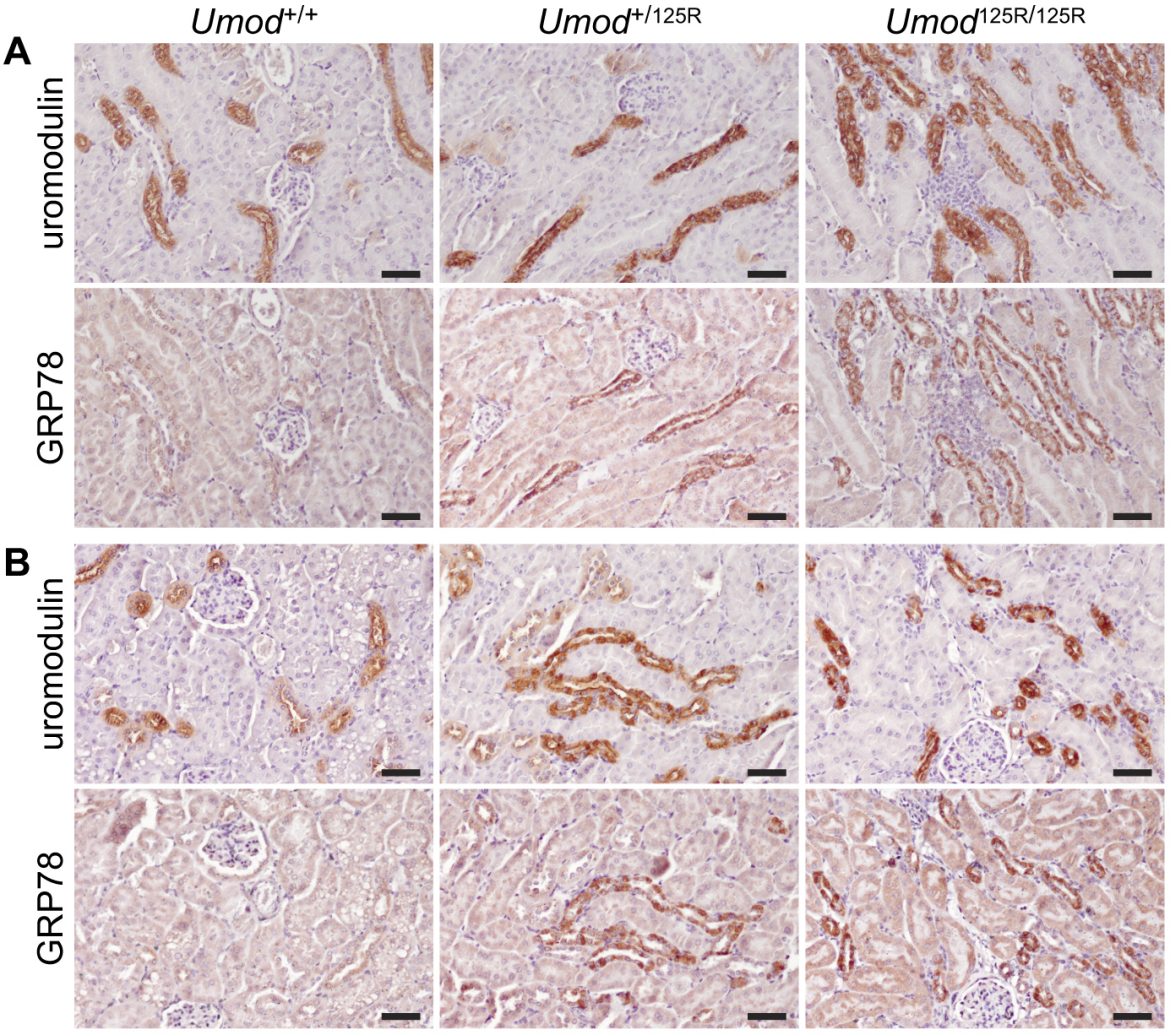
**ER stress in *Umod*^+/+^, *Umod*^+/125R^ and *Umod*^125R/125R^ kidneys.** Immunohistochemical staining of serial sections for uromodulin and GRP78 in (A) 8-week-old and (B) 6-month-old mice, showing increased expression of GRP78 specifically in TAL cells expressing mutant uromodulin in *Umod*^+/125R^ and *Umod*^125R/125R^ kidneys. Nuclei counterstained with hematoxylin; scale bars: 50 µm.

Accumulation of mutant proteins in the ER may cause ER stress, triggering an adaptive pathway called the unfolded protein response (UPR). Members of the chaperone family of GRPs, and in particular GRP78, are upregulated by the UPR in response to ER stress. We therefore used immunohistochemistry of serial kidney sections to investigate GRP78 expression in TAL cells expressing mutant uromodulin. In *Umod*^+/+^ kidneys, GRP78 was expressed at a very low level in non-TAL tubular epithelial cells, and at a slightly elevated level in TAL tubular cells (Fig. 3A,B), a finding that was confirmed in kidneys from an unrelated mouse line (data not shown). However, in kidneys from both 8-week- and 6-month-old *Umod*^+/125R^ and *Umod*^125R/125R^ mice, GRP78 expression was upregulated specifically in TAL cells expressing mutant uromodulin, and not in other tubular segments (Fig. 3A,B).

**Fig. 4.**
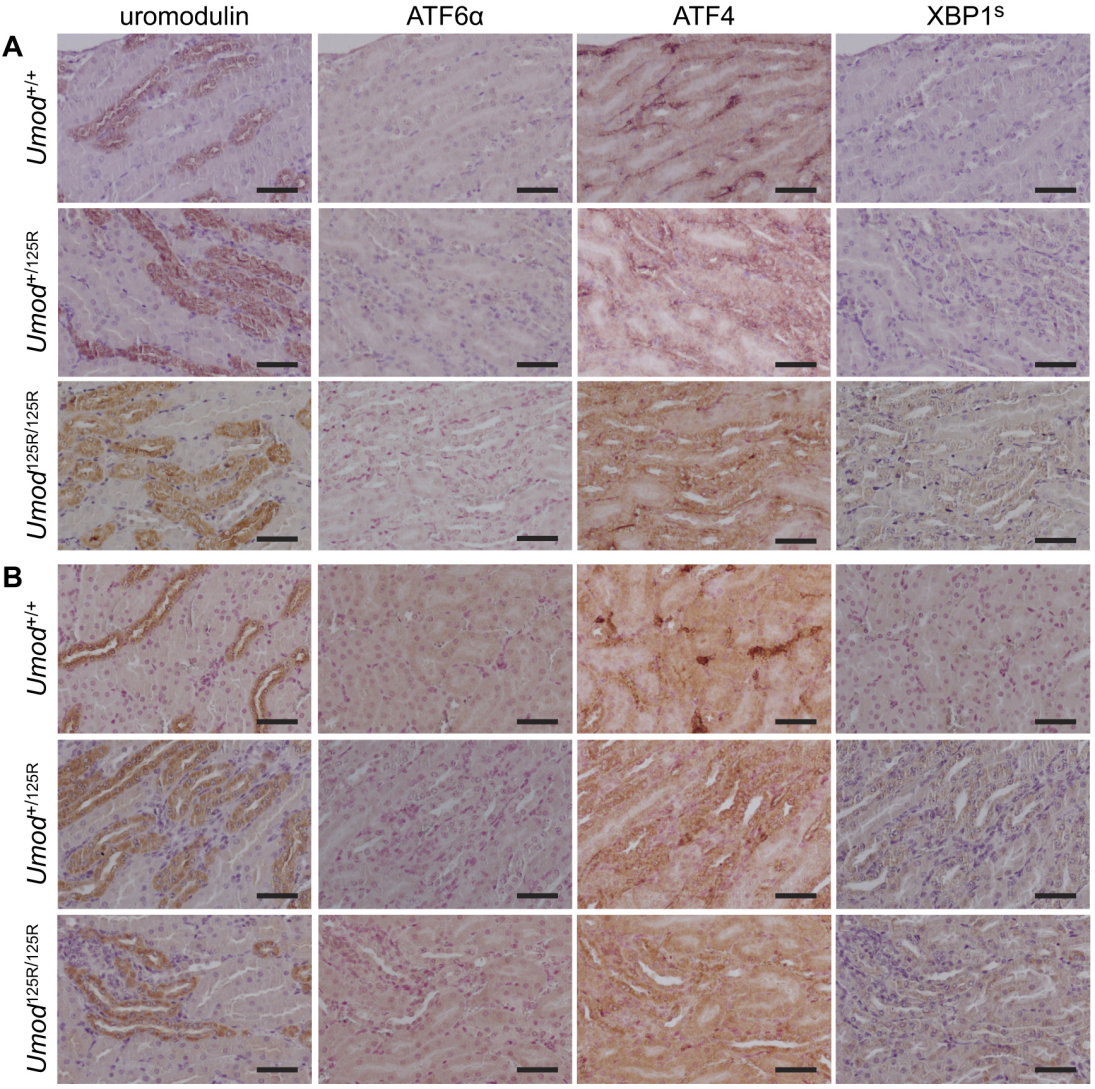
**Analysis of UPR pathways in *Umod*^+/+^, *Umod*^+/125R^ and *Umod*^125R/125R^ kidneys.** Immunohistochemical staining of serial sections for uromodulin, ATF6α, ATF4 and spliced (active) XBP1 (XBP1^S^) in (A) 8-week-old and (B) 6-month-old mice, showing upregulation of ATF4 and XBP1^S^ specifically in TAL cells expressing mutant uromodulin in *Umod*^+/125R^ and *Umod*^125R/125R^ kidneys, but no upregulation of ATF6α. Nuclei counterstained with hematoxylin; scale bars: 50 µm.

We sought to determine which of the three UPR pathways may be active in TAL cells expressing mutant uromodulin, by immunohistochemistry to detect expression of a member of each of the three pathway, which are: ATF6α (ATF6α pathway), spliced (active) XBP1 (XBP1^S^; IRE1 pathway) or ATF4 (PERK pathway). None of the three pathways were upregulated in TAL cells in *Umod*^+/+^ mice when compared to non-TAL cells (Fig. 4A,B). No activation of the ATF6α pathway was detected in *Umod*^+/125R^ or *Umod*^125R/125R^ mice at either 8 weeks or 6 months of age, as there was no increase in nuclear ATF6α expression in TAL cells (Fig. 4A,B). By contrast, both XBP1^S^ and ATF4 were upregulated in TAL cells of *Umod*^+/125R^ and *Umod*^125R/125R^ mice compared to *Umod*^+/+^ littermates at both 8 weeks and 6 months of age (Fig. 4A,B), demonstrating upregulation of the IRE1 and PERK pathways, respectively, in response to retention of mutant uromodulin in the ER.

**Fig. 5.**
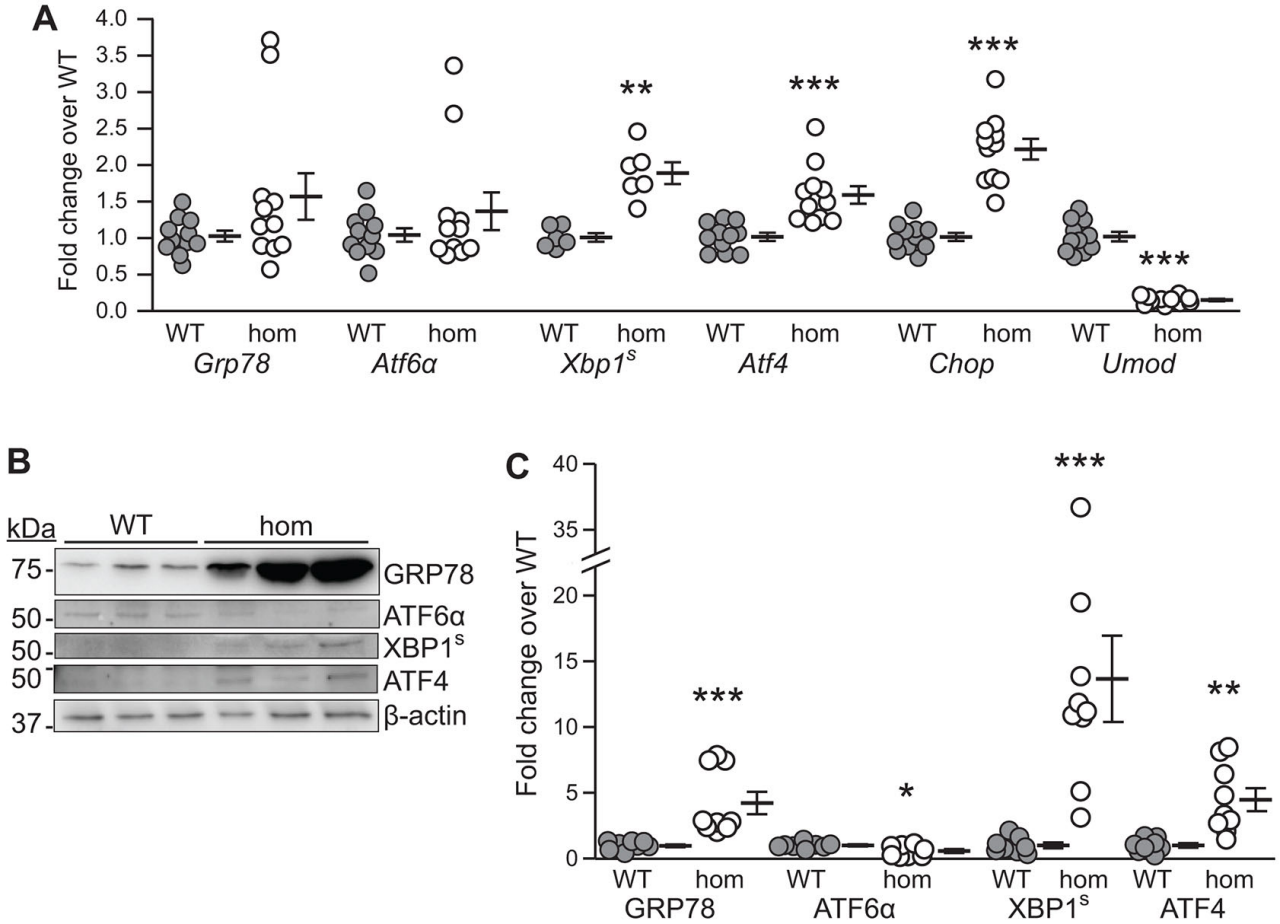
**Expression of ER stress and UPR pathway components in isolated *Umod*^+/+^ (WT) and *Umod*^125R/125R^ (hom) TALs.** (A) qRT-PCR analysis of *Grp78*, *Atf6α*, spliced (active) *Xbp1* (*Xbp1*^S^), *Atf4*, *Chop* and *Umod*. Gene expression changes are relative to *Gapdh* and expressed as a fold change over WT expression (mean set to 1). Individual TAL preparations (*n*=6-11) from 2-4 mice per group are represented as single points; mean±s.e.m. are displayed to the right of each data set; **P<0.01, ***P<0.001 versus WT, using 2-tailed Mann–Whitney *U*-test. (B) Representative western blots and (C) quantification of GRP78, ATF6α, XBP1^S^ and ATF4. Protein was normalized for loading using β-actin and quantification is expressed as a fold change over WT expression (mean set to 1). Individual TAL preparations (*n*=8-11) from 3-4 mice per group are represented as single points; mean±s.e.m. are displayed to the right of each data set; *P<0.05, **P<0.01, ***P<0.001 versus WT, using 2-tailed Mann–Whitney *U*-test.

In order to quantify the upregulation of these ER stress and UPR pathways, we microdissected TALs from *Umod*^+/+^ and *Umod*^125R/125R^ mice. Quantitative real-time PCR (qRT-PCR) analysis for nephron segment-specific genes was undertaken to demonstrate the purity of the obtained TALs (data not shown), followed by qRT-PCR and western blot analyses for UPR components. qRT-PCR analysis showed that *Xbp1*^S^ and *Atf4* were significantly upregulated by ∼1.9-fold (*P*<0.01) and ∼1.6-fold (*P*<0.001), respectively, in *Umod*^125R/125R^ TALs compared to *Umod*^+/+^ TALs, but that *Grp78* and *Atf6α* expression levels were not significantly different (Fig. 5A). However, western blot analysis showed that GRP78 protein was significantly upregulated by ∼4.2-fold (*P*<0.001) in *Umod*^125R/125R^ TALs compared to *Umod*^+/+^ TALs, consistent with regulation of GRP78 expression at the translational level (Gulow et al., 2002) (Fig. 5B,C). Interestingly, ATF6α was significantly downregulated by ∼1.8-fold (*P*<0.05) in *Umod*^125R/125R^ TALs compared to *Umod*^+/+^ TALs (Fig. 5B,C). XBP1^S^ and ATF4 were significantly upregulated by ∼13.6-fold (*P*<0.001) and ∼4.5-fold (*P*<0.01), respectively, in *Umod*^125R/125R^ TALs (Fig. 5B,C), confirming the results obtained by immunohistochemical analysis.

**Fig. 6.**
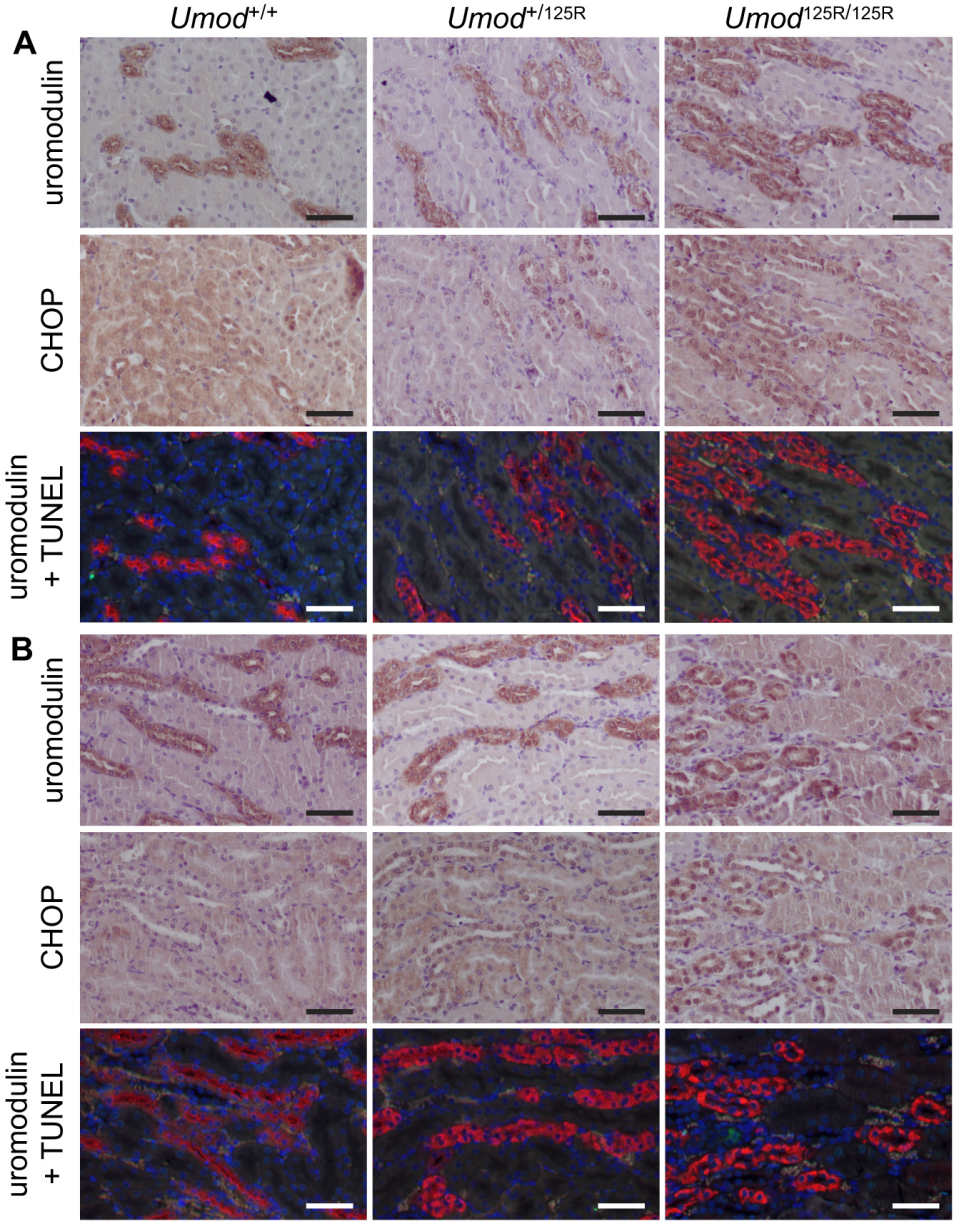
**Analysis of CHOP expression and apoptosis in *Umod*^+/+^, *Umod*^+/125R^ and *Umod*^125R/125R^ kidneys.** Immunohistochemical staining for uromodulin and CHOP, and co-immunofluorescence using TUNEL assay to label apoptotic cells (green) and anti-uromodulin antibody to label TAL cells (red), in serial sections in (A) 8-week-old and (B) 6-month-old mice. CHOP is upregulated in the nuclei of TAL cells expressing mutant uromodulin in *Umod*^+/125R^ and *Umod*^125R/125R^ kidneys, but there is an absence of apoptosis in TAL cells. Nuclei counterstained with hematoxylin (immunohistochemistry) or DAPI (immunofluorescence); scale bars: 50 µm.

Upregulation of the PERK pathway via ATF4 is associated with suppression of general protein translation but induction of expression of specific UPR target genes, such as CCAAT/enhancer-binding protein homologous protein (CHOP) (Hetz, 2012). qRT-PCR analysis of isolated TALs showed that *Umod* mRNA was downregulated by ∼6.6-fold (*P*<0.001) in *Umod*^125R/125R^ TALs compared to *Umod*^+/+^ TALs (Fig. 5A). *Chop* mRNA was upregulated by ∼2.2-fold (*P*<0.001) in *Umod*^125R/125R^ TALs compared to *Umod*^+/+^ TALs (Fig. 5A), and immunohistochemical analysis of serial kidney sections confirmed that CHOP protein was upregulated in TAL cells in *Umod*^+/125R^ and *Umod*^125R/125R^ mice compared to *Umod*^+/+^ littermates at both at 8 weeks and 6 months of age, as shown by accumulation of CHOP in the nuclei of TAL cells (Fig. 6A,B). Increased CHOP expression may lead to apoptosis (Hetz, 2012); however, analysis of kidney sections using terminal deoxynucleotidyl transferase dUTP nick end labeling (TUNEL) assay and co-staining for uromodulin showed that, as in *Umod*^+/+^ littermates, there were no TUNEL-positive TAL cells in *Umod*^+/125R^ and *Umod*^125R/125R^ mouse kidneys at either 8 weeks or 6 months of age (Fig. 6A,B). However, occasional TUNEL-positive cells were detected in other segments, demonstrating that the assay was functional. Thus, mechanisms other than apoptosis are likely responsible for tubular damage in *Umod*^+/125R^ and *Umod*^125R/125R^ mice.

## DISCUSSION

We have generated a mouse model with an ADTKD disease-causing *UMOD* mutation, that displays: defective uric acid excretion; urinary concentrating defects; renal failure; defective uromodulin trafficking, maturation and secretion; renal fibrosis; interstitial immune cell infiltration; and ER stress with upregulation of the UPR in the TAL. This model is representative of ADTKD-*UMOD* patients, who develop hyperuricemia, low FEUA and gout, chronic renal failure, tubulointerstitial nephropathy, glomerulosclerosis and lymphocytic infiltration (Lhotta et al., 1998; Turner et al., 2003). Thus, this mouse provides an *in vivo* model to study mechanisms of renal fibrosis and ER stress.

A major feature of ADTKD-*UMOD* in humans is hyperuricemia, which was not present in *Umod* mutant mice. This is not surprising, as mice express the hepatic enzyme uricase (urate oxidase), which catalyses low solubility uric acid to the high solubility allantoin; uricase has been evolutionarily silenced in humans and other primates (Wu et al., 1992). However, *Umod*^+/125R^ and *Umod*^125R/125R^ mice, similar to ADTKD-*UMOD* patients, still demonstrated significant decreases in both FEUA and 24 hour uric acid excretion. Interestingly, in female *Umod*^125R/125R^ mice, this decreased uric acid excretion was detected after the onset of renal failure, as measured by a significantly increased plasma urea, since 8-week-old *Umod*^125R/125R^ females had an elevated plasma urea, but a similar uric acid excretion compared to *Umod*^+/+^ females. The role of plasma uric acid levels in the pathogenesis of ADTKD-*UMOD* has been the subject of much debate, particularly whether drugs that lower plasma uric acid can slow the progression of ADTKD-*UMOD* in patients (Bleyer and Hart, 2003; Fairbanks et al., 2002, 2004; Puig et al., 2006; Puig and Torres, 2003, 2004). Our model demonstrates that hyperuricemia is a secondary consequence of the other phenotypes of ADTKD-*UMOD* (e.g. decreased uric acid excretion, renal failure and kidney histopathology), and is not required to cause the renal pathophysiology, since our model develops these other phenotypes without hyperuricemia. However, hyperuricemia may help to propagate or enhance the disease progression in humans, and this hypothesis could be tested in our model by using uricase inhibitors.

The mechanism of decreased uric acid excretion is not fully understood, but may be a consequence of the urine concentrating defect. In the normal TAL, the water impermeability is likely due to the presence of uromodulin in a gel-like structure at the apical plasma membrane, which is permeable to ions but not to water (Mattey and Naftalin, 1992). In *Umod*^+/125R^ and *Umod*^125R/125R^ mice, retention of the mutant uromodulin protein in the ER of the TAL cells would decrease the amount of uromodulin at the apical plasma membrane, and may compromise this water impermeability. This may lead to a reduction in the normally high osmolality of the medullary interstitium, thus inhibiting water reabsorption in the collecting duct (CD), and resulting in a more dilute urine (Devuyst et al., 2005). The previously reported *Umod*^−/−^ knock-out mice also have a urine concentrating defect (Bachmann et al., 2005). This defect in urine concentration due to alterations of TAL characteristics may be analogous to patients treated with loop diuretics, such as those with hypertension, who also develop secondary hyperuricemia (Ramsay et al., 1994). Loop diuretics (e.g. furosemide) specifically inhibit the TAL Na^+^:K^+^:2Cl^−^ (NKCC2) transporter, decreasing water reabsorption with a consequent increase in urine volume (Gamba and Friedman, 2009). During loop diuretic treatment, the resulting decrease in extracellular fluid (ECF) volume causes increased sodium reabsorption by the proximal convoluted tubule (PCT), which likely drives increased uric acid reabsorption, hence causing the hyperuricemia which is often associated with these drugs (Weinman et al., 1975). A possible compensatory mechanism for excess water loss has been described in *Umod*^−/−^ knock-out mice by upregulation of several critical TAL ion transporters, including the target of loop diuretics, NKCC2, which is usually activated by uromodulin (Bachmann et al., 2005). Thus, similar mechanisms may be responsible for hyperuricemia in loop diuretic-treated hypertensive patients and ADTKD-*UMOD* patients; this requires further investigation in *Umod*^+/125R^ and *Umod*^125R/125R^ mice.

The urine concentrating defect described in *Umod*^−/−^ knock-out mice (Bachmann et al., 2005) and *Umod*^+/125R^ and *Umod*^125R/125R^ mice in this study is likely due to the absence of uromodulin at the apical plasma membrane of the TAL. However, *Umod*^−/−^ knock-out mice do not develop any histological characteristics of ADTKD-*UMOD*, or renal failure (Raffi et al., 2006). Thus, mutant uromodulin protein must be present to cause the renal pathology associated with ADTKD-*UMOD*, and we have shown that histopathological changes characteristic of human ADTKD-*UMOD* patients, such as interstitial fibrosis and immune cell infiltration, are present in our *Umod*^+/125R^ and *Umod*^125R/125R^ knock-in mice. Uromodulin is the most abundant protein in the urine, and is expressed only by the TAL (Scolari et al., 2015), indicating a high rate of uromodulin expression in TAL cells. Thus, even though cells possess mechanisms to degrade misfolded proteins, such as ERAD, the high expression rate of mutant uromodulin over time may overwhelm such mechanisms. Uromodulin can also stimulate the innate and adaptive immune systems (Säemann et al., 2005b; Su et al., 1997). Thus, it has been postulated that kidney damage may compromise its integrity and allow uromodulin to be exposed to the immune system, which may cause antibody production (Säemann et al., 2005b), as well as inflammation and/or immune cell infiltration, which can result in fibrosis of the kidney (Eddy, 2014).

*Umod*^+/125R^ and *Umod*^125R/125R^ mice have several phenotypic characteristics that are similar to those reported for the C147W uromodulin transgenic (Tg*^Umod^*^C147W^) mouse model, including renal failure, urinary concentrating defects, fibrosis, immune cell infiltration and intracellular uromodulin retention. However, *Umod*^+/125R^ and *Umod*^125R/125R^ mice had a selective defect in renal uric acid handling that was not studied in Tg*^Umod^*^C147W^ transgenic mice (Bernascone et al., 2010), whereas Tg*^Umod^*^C147W^ mice had renal cysts, which were not detected in *Umod*^+/125R^ or *Umod*^125R/125R^ mice (Table S1). The reason for these differences between *Umod*^125R^ mice and Tg*^Umod^*^C147W^ mice remains to be elucidated, but there are several possible explanations. First, these differences may involve strain-specific factors, as the Tg*^Umod^*^C147W^ mice were generated on an FVB background, whilst the *Umod*^125R^ mice were on a C57BL/6 background. Second, the Tg*^Umod^*^C147W^ mice are a transgenic model that express *Umod* at higher levels than wild-type mice without the transgene, whereas, in *Umod*^125R^ mice, the mutant *Umod* directly replaces the wild-type allele. Third, Tg*^Umod^*^C147W^ mice express mutant uromodulin with a different mutation compared to *Umod*^125R^ mice. Patients with different mutations have marked variations in some phenotypic features, such as rate of progression to ESRF (Moskowitz et al., 2013); however, this does not seem to be true of hyperuricemia, which is present in >90% of patients (Williams et al., 2009). The presence or absence of cysts is the most variable phenotype associated with ADTKD-*UMOD* in humans and may only be found in <15% of patients (Williams et al., 2009). Indeed, different members of the same family may have variable cyst formation (Rampoldi et al., 2003; Vylet'al et al., 2006); this variation may be due to other genetic differences or environmental factors rather than to different *UMOD* mutations. Cysts have not been reported in families with either the C126R or C148W mutations (Lhotta et al., 1998; Rampoldi et al., 2003), and the basis for this variation in cyst formation between different families, family members and mouse models requires further investigation. Two ENU-mutagenized mouse models, carrying A227T or C93F uromodulin mutations on the same backgrounds, have recently been described to have differing rates of disease onset and progression, with C93F mutant mice having the more severe phenotype (Kemter et al., 2013). The ENU mutant mice both displayed decreased uric acid excretion that was present in *Umod*^125R^ mice but absent in Tg*^Umod^*^C147W^ mice, tubulocystic changes that were present in Tg*^Umod^*^C147W^ mice but absent in *Umod*^125R^ mice, and glomerulocystic changes that were absent in both Tg*^Umod^*^C147W^ and *Umod*^125R^ mice (Bernascone et al., 2010; Kemter et al., 2013) (Table S1). These differences between the germline *Umod*^125R^, transgenic Tg*^Umod^*^C147W^ and ENU A227T and C93F mice with different mutations highlight the advantages of having multiple mouse models that provide the opportunity of elucidating further the relationships between protein, uromodulin mutation, genetic modifiers, phenotype and mechanism in ADTKD-*UMOD*.

Our results provide mechanistic insights by showing that ER stress is present in TAL cells expressing mutant uromodulin, and that two of the three UPR pathways (PERK and IRE1) are active, leading to upregulation of CHOP. However, this did not lead to apoptosis of TAL cells, demonstrating that other mechanisms may be responsible for TAL damage, and consequent fibrosis and immune cell infiltration, in this model. CHOP is a transcription factor typically thought to upregulate pro-apoptotic pathways; however, it may also be protective under certain conditions in different cell types. For example, mice with autosomal dominant retinitis pigmentosa due to ER retention of mutant rhodopsin have retinal damage that is associated with UPR and CHOP upregulation; however, deletion of CHOP led to increased retinal damage in these mice, suggesting a protective role for CHOP (Nashine et al., 2013). Several studies have implicated ER stress, leading to CHOP expression and apoptosis, in models of renal disease. However, these studies have primarily investigated the renal response to toxins (e.g. high glucose in diabetic nephropathy or high albumin levels to mimic proteinuria), drugs (e.g. acetaminophen) or acute renal injury (e.g. using unilateral ureteric obstruction), or have focused on glomerular injury (Dickhout and Krepinsky, 2009). Relatively little work has been carried out to understand the role of chronic ER stress in renal tubule cells endogenously expressing an ER-retained mutant protein, and our mouse model for ADTKD-*UMOD* will be an important resource for further studies in this area. Active XBP1 protein was also upregulated in TAL cells in *Umod*^125R^ mice, demonstrating upregulation of the IRE1 pathway, and this may also be an adaptive or apoptotic response to ER stress (Ghosh et al., 2014). During prolonged ER stress, PERK signaling is maintained whilst IRE1 signaling is attenuated, which may lead to a switch from adaptive to apoptotic signaling (Lin et al., 2007). Consistent with this hypothesis, prolonged IRE1 signaling enhanced cell proliferation and prevented apoptosis when human embryonic kidney (HEK)293 cells were exposed to the UPR inducers tunicamycin and thapsigargin (Lin et al., 2007, 2009). In *Umod*^125R^ mice, both the PERK and IRE1 pathways appeared to be active even at 6 months of age, and the presence of both of these pathways may explain the absence of the apoptotic response in TAL cells in these mice. Studies of the transgenic Tg*^Umod^*^C147W^ mice also showed an absence of apoptosis by a lack of active caspase-3 staining; thus, apoptosis does not appear to play a significant role in tubule damage in ADTKD-*UMOD* (Bernascone et al., 2010).

In conclusion, we have generated a mouse model for a monogenic cause of renal fibrosis carrying an ADTKD-*UMOD* disease-causing mutation, C125R, in the endogenous mouse *Umod* gene. *Umod*^+/125R^ and *Umod*^125R/125R^ mice demonstrate several of the phenotypes of human ADTKD-*UMOD* patients, including progressive renal failure, urine concentrating defects, decreased uric acid and uromodulin excretion, retention of uromodulin in the ER, and pathological changes in the kidney such as fibrosis, immune cell infiltration and ER stress. The PERK and IRE1 UPR pathways were found to be specifically upregulated in TAL cells of *Umod*^+/125R^ and *Umod*^125R/125R^ mice, which was associated with upregulation of CHOP; however, this did not lead to apoptosis. This novel knock-in mouse model will be a valuable resource to study the mechanisms of pathogenesis and progression of renal fibrosis, ESRF and ADTKD-*UMOD*, and to test novel therapeutic agents for these diseases.

## MATERIALS AND METHODS

### Generation of knock-in targeting vector and mutant mice

Exon 3 of mouse *Umod* was amplified by PCR and used to screen a mouse PAC library (MRC Geneservice, Cambridge, UK). A PAC containing mouse *Umod* was then used as a template in a long-range PCR reaction to amplify an ∼7.6 kb product between the 5′ upstream region and intron 5 of *Umod*. Exon and intron-exon boundary sequences were verified by direct DNA sequencing as described (Nesbit et al., 2004). After insertion into pGEM-T (Promega, Southampton, UK), site-directed mutagenesis (SDM) was performed to introduce the C125R mutation as described (Nesbit et al., 2004); during this procedure, a novel *Bso*BI restriction site was also generated. SDM was also used to introduce a *Hin*dIII site into intron 2. Following each round of SDM, the sequences of exons and intron-exon boundaries were verified by direct DNA sequencing. A neomycin resistance (Neo^R^) cassette flanked by two *loxP* sites was excised from plasmid pmmneoflox8 and cloned into intron 2 of *Umod* using *Xba*I and *Hin*dIII. Plasmid pBSTK, containing a thymidine kinase (TK) cassette, was mutagenized by SDM to introduce a *Sfi*I restriction site, and the TK cassette was inserted into the targeting vector at the 5′ end of the *Umod* gene using *Sfi*I and *Sac*II (Fig. S1). The vector was linearized using *Sbf*I, electroporated into the R1 embryonic stem (ES) cell line, and cells selected using 300 µg/ml G418 (Geneticin, Gibco, Paisley, UK) and 0.2 µM gancyclovir (Calbiochem, Manchester, UK). Cells in which homologous recombination had occurred at both the 5′ and 3′ arms were identified by PCR/restriction digest and Southern blot analysis (Harding et al., 2009) as follows: 5′ PCR: forward primer in the 5′ region outside the vector and reverse primer within the Neo^R^ cassette generated a 2.8 kb product in recombined clones only; 3′ PCR: forward primer within exon 3 and reverse primer within intron 5 (outside the vector) generated a 5.3 kb fragment in all clones, which, when digested with *Bso*BI, generated an extra fragment in recombined clones; 5′ Southern blot: digestion with *Pfl*FI and a probe within the 5′ upstream region detected an ∼11.6 kb *Umod* wild-type fragment and an ∼7.4 kb *Umod* mutant fragment; 3′ Southern blot: digestion with *Spe*I and a probe corresponding to exon 6 of *Umod* detected an ∼8.3 kb *Umod* wild-type fragment and an ∼9.6 kb *Umod* mutant fragment (Fig. 1A). ES cells heterozygous for the mutation were injected into C57BL/6 blastocysts at embryonic day 2.5, which were transferred into the uterus of pseudopregnant C57BL/6 females. Male chimeras were mated with C57BL/6 females and the offspring tested for germline transmission by extraction of DNA from ear biopsy material in DNA extraction buffer (50 mM Tris pH 8.5, 1 mM EDTA, 0.5% Tween-20, with 50 µg Proteinase K), followed by PCR and *Bso*BI digestion. The Neo^R^ cassette was excised between *loxP* sites by mating with β-actin-Cre mice, and the removal of the Neo^R^ cassette was verified by PCR assay. Mice were backcrossed to the C57BL/6J background strain and mice from backcross (BC)3-BC5 were used for studies in 6-month-old mice, and from BC4-BC7 for studies in 8-week-old mice. Animals were maintained according to local welfare guidelines, and all procedures were carried out under UK Home Office personal and project license restrictions.

### Metabolic cage analysis

Eight-week- and 6-month-old *Umod*^+/+^, *Umod*^+/125R^ and *Umod*^125R/125R^ littermates were investigated in individual metabolic cages (Techniplast) for 24 h with free access to food and water. Body weight, water intake and urine output were measured and urine was collected under mineral oil. Mice were euthanized by terminal anesthesia, blood collected via the jugular vein, and plasma obtained by centrifugation at 800 ***g*** for 10 min. Plasma and urine biochemical analysis was performed on a Beckman Coulter AU680 semi-automated clinical biochemistry analyzer as described (Piret et al., 2012), and plasma calcium was corrected for albumin (Corr.Ca) using the formula: Corr.Ca=Ca(mmol/l)−[(Alb(g/l)−30)×0.017] (Piret et al., 2012). Osmolalities were measured using a Model 3320 Osmometer (Advanced Instruments Inc., Norwood, MA, USA) and urinary pH was measured using a Microprocessor pH meter (Hanna Instruments, Leighton Buzzard, UK).

### Western blot analysis of whole kidney lysates and urine

Uromodulin maturation and excretion in *Umod*^+/+^, *Umod*^+/125R^ and *Umod*^125R/125R^ mutant mice were studied by western blot analysis of kidney lysates and urine samples. Kidneys were homogenized in β-octylglucoside lysis buffer [20 mM Tris-HCl pH 8; 150 mM NaCl; 60 mM β-octylglucoside (Bernascone et al., 2010)] with a protease inhibitor cocktail (Roche, Burgess Hill, UK). Insoluble material was removed by centrifugation, total protein concentration of the soluble fraction determined using the micro BCA Protein Assay (Pierce, UK) (Bernascone et al., 2010; Williams et al., 2009), and lysates loaded at 20-50 µg total protein per lane. Urine samples were normalized to urinary creatinine such that, for *Umod*^+/+^ and *Umod*^+/125R^ samples, a urine volume corresponding to 0.5 nmol creatinine was loaded and, for *Umod*^125R/125R^ samples, a urine volume corresponding to 10 nmol was loaded. Western blotting and densitometric analysis were performed as previously described, using sheep anti-uromucoid antibody (Biodesign International, Saco, ME, USA), or rabbit anti-tubulin antibody (Abcam ab15246, 1:500), followed by horseradish peroxidase (HRP)-conjugated mouse anti-sheep (Sigma, Poole, UK) or HRP-conjugated goat anti-rabbit (Bio-Rad, Watford, UK) antibodies, respectively (Williams et al., 2009). To control for variances between blots of urinary uromodulin, at least two *Umod*^+/+^ samples were loaded on each gel, a standard ‘remove background’ tool was used prior to quantification by densitometry, and mean densities of *Umod*^+/+^ bands were set to 100% for comparison with other bands on the same blot.

### Histological and immunohistochemical analysis

Kidneys were dissected and fixed in 10% neutral buffered formalin for 24 h, then dehydrated, paraffin embedded and sectioned at 5 µm thickness. Trichrome staining was carried out according to standard protocols. For immunohistochemical labeling, sections were rehydrated, then subjected to antigen unmasking either by incubation in 20 µg/ml proteinase K for 5-15 min, or by heat-mediated antigen retrieval in either pH 6, pH 9 or pH 10 buffers. Sections were blocked in pre-immune serum, and probed with the following primary antibodies: sheep anti-uromucoid (Biodesign International, 1:500); rabbit anti-calnexin (Abcam ab22595, 1:50); rabbit anti-CD3 (Abcam ab16669, 1:50); rat anti-F4/80 (Serotec clone A3-1, 1:50); rabbit anti-GRP78 (Abcam ab21685, 1:1000); rabbit anti-ATF4 (Abcam ab105383, 1:500); rabbit anti-ATF6α [Santa Cruz Biotechnology (Santa Cruz, CA, USA) sc-22799, 1:50]; goat anti-XBP1 (spliced) (Abcam ab85546, 1:100); or rabbit anti-CHOP (Santa Cruz sc-575, 1:50). Secondary detection was achieved by: HRP-conjugated donkey anti-sheep antibody (Jackson ImmunoResearch, 1:500) visualized using the 3,3′-Diaminobenzidine (DAB) reaction kit (Vector Labs, Peterborough, UK) for uromodulin; the DAKO Envision+ System DAB kit for rabbit primary antibodies; the Santa Cruz ABC staining system for goat primary antibodies; or fluorescently conjugated donkey secondary antibodies (Jackson ImmunoResearch or Invitrogen) for dual-labeling. TUNEL assay was performed using the ApopTag Fluorescein kit (Millipore, Hertfordshire, UK) according to the manufacturer's instructions. DAB-stained slides were counterstained with hematoxylin, and fluorescently labeled slides were counterstained with 4′,6-diamidino-2-phenylindole (DAPI). Slides were examined by light or UV microscopy using an Eclipse E400 microscope (Nikon, Japan), and images were captured using a DXM1200C digital camera, with an original magnification of 20× or 40×, and NIS-Elements BR2.30 software (both Nikon). Immunohistochemistry was performed on 2 kidney sections from each of 4 mice per group.

### Isolation of mouse TALs, and qRT-PCR and western blot analyses

Mouse TALs were isolated following a previously described protocol (Glaudemans et al., 2014) with minor modifications. Briefly, 2- to 3-month-old male *Umod*^+/+^ and *Umod*^125R/125R^ mice were sacrificed by cervical dislocation. Kidneys were removed, decapsulated and placed in ice-cold dissection solution (Hanks balanced salt solution with 15 mM HEPES, 10 mM D-glucose, 5 mM glycine, 1 mM L-alanine, adjusted to 325 mOsm/kgH_2_O with mannitol and pH 7.4 with NaOH). Each kidney was cut into 2 equal parts by a midsagittal section and then into thin transverse slices that were further cut into radial corticomedullary stripes. Kidney pieces were digested for 30 min in dissection solution supplemented with 245 U/ml type 2 collagenase (Worthington Biochemical Corporation, Lakewood, NJ, USA) and 96 µg/ml soybean trypsin inhibitor (Sigma-Aldrich) at 37°C on a shaker. The supernatant containing small kidney fragments and isolated tubules was sieved through 250 µm and 50 µm filters (Sefar AG, Heiden, Switzerland) and tubules retained on the 50 µm filter were collected in 37°C dissection solution supplemented with 1% BSA (Sigma-Aldrich) and 96 µg/ml soybean trypsin inhibitor (Sigma-Aldrich). Remaining undigested kidney fragments were digested for 10 min in fresh collagenase solution. TALs were manually selected under a light microscope using a glass pipet connected to a micromanipulator (Narishige International, London, UK). Total RNA was extracted from microdissected TALs using RNAqueous^®^ Total RNA Isolation Kit (Invitrogen, MA, USA), following the manufacturer's protocol, and DNAseI treatment performed. The reverse transcriptase (RT) reaction was performed using 1 µg of RNA and the iScript™ cDNA Synthesis Kit (Bio-Rad). Changes in mRNA levels of target genes were determined by relative qRT-PCR with a CFX96™ Real-Time PCR Detection System (Bio-Rad) using iQ™ SYBR Green Supermix (Bio-Rad) detection of single PCR product accumulation in duplicate. Specific primers were designed using Primer3. PCR conditions were 95°C for 3 min followed by 40 cycles of 15 s at 95°C, 30 s at 60°C. PCR products were sequenced using the BigDye Terminator Kit (Perkin Elmer Applied Biosystems, MA, USA), purified using the multiScreen SEQ_384_ Filter Plate (Millipore) and Sephadex G-50 DNA Grade Fine (Amersham Biosciences, NJ, USA), and analyzed on an ABI3100 capillary sequencer (Perkin Elmer Applied Biosystems). The efficiency of each set of primers was determined by dilution curves. Glyceraldehyde 3-phosphate dehydrogenase (*Gapdh*) was used routinely as a reference gene. Relative changes in target gene/*Gapdh* mRNA ratio were determined by the formula: 2^ΔΔct^. For western blot analysis, 50-70 TALs were pooled and directly transferred in 4× Laemmli sample buffer (Bio-Rad) supplemented with protease inhibitors (Roche, Basel, Switzerland) and 60 mg/ml dithiothreitol (AppliChem), and boiled at 95°C for 5 min. Western blotting and densitometric analysis were performed using antibodies against ATF4, ATF6α, GRP78 and spliced XBP1, as above.

### Statistical analysis

Statistical analyses were performed using Student's unpaired *t*-test or the Mann–Whitney *U*-test, where appropriate.

### Study approval

All animal studies were approved by the University of Oxford Ethical Review Committee and were licensed under the Animals (Scientific Procedures) Act 1986, issued by the United Kingdom Government Home Office Department.

## References

[DMM029488C1] Adam J., Bollée G., Fougeray S., Noël L.-H., Antignac C., Knebelman B. and Pallet N. (2012). Endoplasmic reticulum stress in UMOD-related kidney disease: a human pathologic study. *Am. J. Kidney Dis.* 59, 117-121. 10.1053/j.ajkd.2011.08.014 21978600

[DMM029488C2] Bachmann S., Mutig K., Bates J., Welker P., Geist B., Gross V., Luft F. C., Alenina N., Bader M., Thiele B. J. et al. (2005). Renal effects of Tamm-Horsfall protein (uromodulin) deficiency in mice. *Am. J. Physiol. Renal Physiol.* 288, F559-F567. 10.1152/ajprenal.00143.2004 15522986

[DMM029488C3] Bates J. M., Raffi H. M., Prasadan K., Mascarenhas R., Laszik Z., Maeda N., Hultgren S. J. and Kumar S. (2004). Tamm-Horsfall protein knockout mice are more prone to urinary tract infection: rapid communication. *Kidney Int.* 65, 791-797. 10.1111/j.1523-1755.2004.00452.x 14871399

[DMM029488C4] Bernascone I., Vavassori S., Di Pentima A., Santambrogio S., Lamorte G., Amoroso A., Scolari F., Ghiggeri G. M., Casari G., Polishchuk R. et al. (2006). Defective intracellular trafficking of uromodulin mutant isoforms. *Traffic* 7, 1567-1579. 10.1111/j.1600-0854.2006.00481.x 17010121

[DMM029488C5] Bernascone I., Janas S., Ikehata M., Trudu M., Corbelli A., Schaeffer C., Rastaldi M. P., Devuyst O. and Rampoldi L. (2010). A transgenic mouse model for uromodulin-associated kidney diseases shows specific tubulo-interstitial damage, urinary concentrating defect and renal failure. *Hum. Mol. Genet.* 19, 2998-3010. 10.1093/hmg/ddq205 20472742

[DMM029488C6] Bleyer A. J. and Hart T. C. (2003). Familial juvenile hyperuricaemic nephropathy. *Q. J. Med.* 96, 867-868. 10.1093/qjmed/hcg141 14566042

[DMM029488C7] Bokhove M., Nishimura K., Brunati M., Han L., de Sanctis D., Rampoldi L. and Jovine L. (2016). A structured interdomain linker directs self-polymerization of human uromodulin. *Proc. Natl. Acad. Sci. USA* 113, 1552-1557. 10.1073/pnas.1519803113 26811476 PMC4760807

[DMM029488C8] Choi S. W., Ryu O. H., Choi S. J., Song I. S., Bleyer A. J. and Hart T. C. (2005). Mutant tamm-horsfall glycoprotein accumulation in endoplasmic reticulum induces apoptosis reversed by colchicine and sodium 4-phenylbutyrate. *J. Am. Soc. Nephrol.* 16, 3006-3014. 10.1681/ASN.2005050461 16135773

[DMM029488C9] Dahan K., Devuyst O., Smaers M., Vertommen D., Loute G., Poux J. M., Viron B., Jacquot C., Gagnadoux M. F., Chauveau D. et al. (2003). A cluster of mutations in the UMOD gene causes familial juvenile hyperuricemic nephropathy with abnormal expression of uromodulin. *J. Am. Soc. Nephrol.* 14, 2883-2893. 10.1097/01.ASN.0000092147.83480.B5 14569098

[DMM029488C10] Darisipudi M. N., Thomasova D., Mulay S. R., Brech D., Noessner E., Liapis H. and Anders H.-J. (2012). Uromodulin triggers IL-1beta-dependent innate immunity via the NLRP3 inflammasome. *J. Am. Soc. Nephrol.* 23, 1783-1789. 10.1681/ASN.2012040338 22997256 PMC3482735

[DMM029488C11] Devuyst O., Dahan K. and Pirson Y. (2005). Tamm-Horsfall protein or uromodulin: new ideas about an old molecule. *Nephrol. Dial. Transplant.* 20, 1290-1294. 10.1093/ndt/gfh851 15840660

[DMM029488C12] Dickhout J. G. and Krepinsky J. C. (2009). Endoplasmic reticulum stress and renal disease. *Antioxid. Redox Signal.* 11, 2341-2352. 10.1089/ars.2009.2705 19508129

[DMM029488C13] Eckardt K.-U., Alper S. L., Antignac C., Bleyer A. J., Chauveau D., Dahan K., Deltas C., Hosking A., Kmoch S., Rampoldi L. et al. (2015). Autosomal dominant tubulointerstitial kidney disease: diagnosis, classification, and management–A KDIGO consensus report. *Kidney Int.* 88, 676-683. 10.1038/ki.2015.28 25738250

[DMM029488C14] Eddy A. A. (2014). Overview of the cellular and molecular basis of kidney fibrosis. *Kidney Int. Suppl.* 4, 2-8. 10.1038/kisup.2014.2 PMC4220516 25401038

[DMM029488C15] Fairbanks L. D., Cameron J. S., Venkat-Raman G., Rigden S. P., Rees L., Van T. H. W., Mansell M., Pattison J., Goldsmith D. J. and Simmonds H. A. (2002). Early treatment with allopurinol in familial juvenile hyerpuricaemic nephropathy (FJHN) ameliorates the long-term progression of renal disease. *Q. J. Med.* 95, 597-607. 10.1093/qjmed/95.9.597 12205338

[DMM029488C16] Fairbanks L. D., Marinaki A. M., Simmonds H. A. and Cameron J. S. (2004). Familial juvenile hyperuricaemic nephropathy. *Q. J. Med.* 97, 106-107. 10.1093/qjmed/hch021 14747627

[DMM029488C17] Gamba G. and Friedman P. A. (2009). Thick ascending limb: the Na(+):K (+):2Cl (-) co-transporter, NKCC2, and the calcium-sensing receptor, CaSR. *Pflugers Archiv* 458, 61-76. 10.1007/s00424-008-0607-1 18982348 PMC3584568

[DMM029488C18] Ghosh R., Wang L., Wang E. S., Perera B. G. K., Igbaria A., Morita S., Prado K., Thamsen M., Caswell D., Macias H. et al. (2014). Allosteric inhibition of the IRE1alpha RNase preserves cell viability and function during endoplasmic reticulum stress. *Cell* 158, 534-548. 10.1016/j.cell.2014.07.002 25018104 PMC4244221

[DMM029488C19] Glaudemans B., Terryn S., Gölz N., Brunati M., Cattaneo A., Bachi A., Al-Qusairi L., Ziegler U., Staub O., Rampoldi L. et al. (2014). A primary culture system of mouse thick ascending limb cells with preserved function and uromodulin processing. *Pflugers Archiv.* 466, 343-356. 10.1007/s00424-013-1321-1 23887378

[DMM029488C20] Gulow K., Bienert D. and Haas I. G. (2002). BiP is feed-back regulated by control of protein translation efficiency. *J. Cell Sci.* 115, 2443-2452. 12006628 10.1242/jcs.115.11.2443

[DMM029488C21] Harding B., Lemos M. C., Reed A. A. C., Walls G. V., Jeyabalan J., Bowl M. R., Tateossian H., Sullivan N., Hough T., Fraser W. D. et al. (2009). Multiple endocrine neoplasia type 1 knockout mice develop parathyroid, pancreatic, pituitary and adrenal tumours with hypercalcaemia, hypophosphataemia and hypercorticosteronaemia. *Endocr. Relat. Cancer* 16, 1313-1327. 10.1677/ERC-09-0082 19620250 PMC4439740

[DMM029488C22] Hart T. C., Gorry M. C., Hart P. S., Woodard A. S., Shihabi Z., Sandhu J., Shirts B., Xu L., Zhu H., Barmada M. M. et al. (2002). Mutations of the UMOD gene are responsible for medullary cystic kidney disease 2 and familial juvenile hyperuricaemic nephropathy. *J. Med. Genet.* 39, 882-892. 10.1136/jmg.39.12.882 12471200 PMC1757206

[DMM029488C23] Hetz C. (2012). The unfolded protein response: controlling cell fate decisions under ER stress and beyond. *Nat. Rev. Mol. Cell Biol.* 13, 89-102. 10.1038/nrm3270 22251901

[DMM029488C24] Jennings P., Aydin S., Kotanko P., Lechner J., Lhotta K., Williams S., Thakker R. V. and Pfaller W. (2007). Membrane targeting and secretion of mutant uromodulin in familial juvenile hyperuricemic nephropathy. *J. Am. Soc. Nephrol.* 18, 264-273. 10.1681/ASN.2006020158 17151335

[DMM029488C25] Kemter E., Rathkolb B., Rozman J., Hans W., Schrewe A., Landbrecht C., Klaften M., Ivandic B., Fuchs H., Gailus-Durner V. et al. (2009). Novel missense mutation of uromodulin in mice causes renal dysfunction with alterations in urea handling, energy, and bone metabolism. *Am. J. Physiol. Renal Physiol.* 297, F1391-F1398. 10.1152/ajprenal.00261.2009 19692485

[DMM029488C26] Kemter E., Prueckl P., Sklenak S., Rathkolb B., Habermann F. A., Hans W., Gailus-Durner V., Fuchs H., Hrabe de Angelis M., Wolf E. et al. (2013). Type of uromodulin mutation and allelic status influence onset and severity of uromodulin-associated kidney disease in mice. *Hum. Mol. Genet.* 22, 4148-4163. 10.1093/hmg/ddt263 23748428

[DMM029488C27] Lhotta K., Gruber J., Sgonc R., Fend F. and König P. (1998). Apoptosis of tubular epithelial cells in familial juvenile gouty nephropathy. *Nephron* 79, 340-344. 10.1159/000045060 9678437

[DMM029488C28] Lin J. H., Li H., Yasumura D., Cohen H. R., Zhang C., Panning B., Shokat K. M., LaVail M. M. and Walter P. (2007). IRE1 signaling affects cell fate during the unfolded protein response. *Science* 318, 944-949. 10.1126/science.1146361 17991856 PMC3670588

[DMM029488C29] Lin J. H., Li H., Zhang Y., Ron D. and Walter P. (2009). Divergent effects of PERK and IRE1 signaling on cell viability. *PLoS ONE* 4, e4170 10.1371/journal.pone.0004170 19137072 PMC2614882

[DMM029488C30] Liu Y., Mo L., Goldfarb D. S., Evan A. P., Liang F., Khan S. R., Lieske J. C. and Wu X.-R. (2010). Progressive renal papillary calcification and ureteral stone formation in mice deficient for Tamm-Horsfall protein. *Am. J. Physiol. Renal Physiol* 299, F469-F478. 10.1152/ajprenal.00243.2010 20591941 PMC2944300

[DMM029488C31] Mattey M. and Naftalin L. (1992). Mechanoelectrical transduction, ion movement and water stasis in uromodulin. *Experientia* 48, 975-980. 10.1007/BF01919145 1426148

[DMM029488C32] Mo L., Huang H.-Y., Zhu X.-H., Shapiro E., Hasty D. L. and Wu X.-R. (2004a). Tamm-Horsfall protein is a critical renal defense factor protecting against calcium oxalate crystal formation. *Kidney Int.* 66, 1159-1166. 10.1111/j.1523-1755.2004.00867.x 15327412

[DMM029488C33] Mo L., Zhu X. H., Huang H. Y., Shapiro E., Hasty D. L. and Wu X. R. (2004b). Ablation of the Tamm-Horsfall protein gene increases susceptibility of mice to bladder colonization by type 1-fimbriated Escherichia coli. *Am. J. Physiol. Renal Physiol.* 286, F795-F802. 10.1152/ajprenal.00357.2003 14665435

[DMM029488C34] Mo L., Liaw L., Evan A. P., Sommer A. J., Lieske J. C. and Wu X.-R. (2007). Renal calcinosis and stone formation in mice lacking osteopontin, Tamm-Horsfall protein, or both. *Am. J. Physiol. Endocrinol. Metab.* 293, F1935-F1943. 10.1152/ajprenal.00383.2007 17898038

[DMM029488C35] Moskowitz J. L., Piret S. E., Lhotta K., Kitzler T. M., Tashman A. P., Velez E., Thakker R. V. and Kotanko P. (2013). Association between genotype and phenotype in uromodulin-associated kidney disease. *Clin. J. Am. Soc. Nephrol.* 8, 1349-1357. 10.2215/CJN.11151012 23723338 PMC3731914

[DMM029488C36] Mutig K., Kahl T., Saritas T., Godes M., Persson P., Bates J., Raffi H., Rampoldi L., Uchida S., Hille C. et al. (2011). Activation of the bumetanide-sensitive Na+,K+,2Cl- cotransporter (NKCC2) is facilitated by Tamm-Horsfall protein in a chloride-sensitive manner. *J. Biol. Chem.* 286, 30200-30210. 10.1074/jbc.M111.222968 21737451 PMC3191059

[DMM029488C37] Nashine S., Bhootada Y., Lewin A. S. and Gorbatyuk M. (2013). Ablation of C/EBP homologous protein does not protect T17M RHO mice from retinal degeneration. *PLoS ONE* 8, e63205 10.1371/journal.pone.0063205 23646198 PMC3640035

[DMM029488C38] Nasr S. H., Lucia J. P., Galgano S. J., Markowitz G. S. and D'Agati V. D. (2008). Uromodulin storage disease. *Kidney Int.* 73, 971-976. 10.1038/sj.ki.5002679 18004297

[DMM029488C39] Nesbit M. A., Bowl M. R., Harding B., Ali A., Ayala A., Crowe C., Dobbie A., Hampson G., Holdaway I., Levine M. A. et al. (2004). Characterization of GATA3 mutations in the hypoparathyroidism, deafness, and renal dysplasia (HDR) syndrome. *J. Biol. Chem.* 279, 22624-22634. 10.1074/jbc.M401797200 14985365

[DMM029488C40] Piret S. E., Esapa C. T., Gorvin C. M., Head R., Loh N. Y., Devuyst O., Thomas G., Brown S. D. M., Brown M., Croucher P. et al. (2012). A mouse model of early-onset renal failure due to a xanthine dehydrogenase nonsense mutation. *PLoS ONE* 7, e45217 10.1371/journal.pone.0045217 23024809 PMC3443222

[DMM029488C41] Puig J. G. and Torres R. J. (2003). Familial juvenile hyperuricaemic nephropathy. *Q. J. Med.* 96, 172-173. 10.1093/qjmed/hcg022b 12589017

[DMM029488C42] Puig J. G. and Torres R. J. (2004). Familial juvenile hyperuricaemic nephropathy. *Q. J. Med.* 97, 457-458. 10.1093/qjmed/hch079 15208434

[DMM029488C43] Puig J. G., Prior C., Martínez-Ara J. and Torres R. J. (2006). Familial nephropathy associated with hyperuricemia in Spain: our experience with 3 families harbouring a UMOD mutation. *Nucleosides Nucleotides Nucleic Acids* 25, 1295-1300. 10.1080/15257770600894766 17065110

[DMM029488C44] Raffi H. S., Bates J. M. Jr, Laszik Z. and Kumar S. (2005). Tamm-Horsfall protein acts as a general host-defense factor against bacterial cystitis. *Am. J. Nephrol.* 25, 570-578. 10.1159/000088990 16244464

[DMM029488C45] Raffi H., Bates J. M., Laszik Z. and Kumar S. (2006). Tamm-Horsfall protein knockout mice do not develop medullary cystic kidney disease. *Kidney Int.* 69, 1914-1915. 10.1038/sj.ki.5000411 16688192

[DMM029488C46] Raffi H. S., Bates J. M. Jr., Laszik Z. and Kumar S. (2009). Tamm-Horsfall protein protects against urinary tract infection by proteus mirabilis. *J. Urol.* 181, 2332-2338. 10.1016/j.juro.2009.01.014 19303096 PMC2930622

[DMM029488C47] Rampoldi L., Caridi G., Santon D., Boaretto F., Bernascone I., Lamorte G., Tardanico R., Dagnino M., Colussi G., Scolari F. et al. (2003). Allelism of MCKD, FJHN and GCKD caused by impairment of uromodulin export dynamics. *Hum. Mol. Genet.* 12, 3369-3384. 10.1093/hmg/ddg353 14570709

[DMM029488C48] Rampoldi L., Scolari F., Amoroso A., Ghiggeri G. M. and Devuyst O. (2011). The rediscovery of uromodulin (Tamm-Horsfall protein): from tubulointerstitial nephropathy to chronic kidney disease. *Kidney Int.* 80, 338-347. 10.1038/ki.2011.134 21654721

[DMM029488C49] Ramsay L. E., Yeo W. W. and Jackson P. R. (1994). Metabolic effects of diuretics. *Cardiology* 84 Suppl. 2, 48-56. 10.1159/000176457 7954546

[DMM029488C50] Renigunta A., Renigunta V., Saritas T., Decher N., Mutig K. and Waldegger S. (2011). Tamm-Horsfall glycoprotein interacts with renal outer medullary potassium channel ROMK2 and regulates its function. *J. Biol. Chem.* 286, 2224-2235. 10.1074/jbc.M110.149880 21081491 PMC3023518

[DMM029488C51] Renkema K. Y., Velic A., Dijkman H. B., Verkaart S., van der Kemp A. W., Nowik M., Timmermans K., Doucet A., Wagner C. A., Bindels R. J. et al. (2009). The calcium-sensing receptor promotes urinary acidification to prevent nephrolithiasis. *J. Am. Soc. Nephrol.* 20, 1705-1713. 10.1681/ASN.2008111195 19470676 PMC2723980

[DMM029488C52] Säemann M. D., Weichhart T., Horl W. H. and Zlabinger G. J. (2005a). Tamm-Horsfall protein: a multilayered defence molecule against urinary tract infection. *Eur. J. Clin. Invest.* 35, 227-235. 10.1111/j.1365-2362.2005.01483.x 15816991

[DMM029488C53] Säemann M. D., Weichhart T., Zeyda M., Staffler G., Schunn M., Stuhlmeier K. M., Sobanov Y., Stulnig T. M., Akira S., von Gabain A. et al. (2005b). Tamm-Horsfall glycoprotein links innate immune cell activation with adaptive immunity via a Toll-like receptor-4-dependent mechanism. *J. Clin. Invest.* 115, 468-475. 10.1172/JCI200522720 15650774 PMC544039

[DMM029488C54] Scolari F., Caridi G., Rampoldi L., Tardanico R., Izzi C., Pirulli D., Amoroso A., Casari G. and Ghiggeri G. M. (2004). Uromodulin storage diseases: clinical aspects and mechanisms. *Am. J. Kidney Dis.* 44, 987-999. 10.1053/j.ajkd.2004.08.021 15558519

[DMM029488C55] Scolari F., Izzi C. and Ghiggeri G. M. (2015). Uromodulin: from monogenic to multifactorial diseases. *Nephrol. Dial. Transplant.* 30, 1250-1256. 10.1093/ndt/gfu300 25228753

[DMM029488C56] Su S. J., Chang K. L., Lin T. M., Huang Y. H. and Yeh T. M. (1997). Uromodulin and Tamm-Horsfall protein induce human monocytes to secrete TNF and express tissue factor. *J. Immunol.* 158, 3449-3456. 9120306

[DMM029488C57] Takiue Y., Hosoyamada M., Yokoo T., Kimura M., Ochiai M., Kaneko K., Ichida K., Hosoya T. and Shibasaki T. (2008a). Production and characterization of transgenic mice harboring mutant human UMOD gene. *Nucleosides Nucleotides Nucleic Acids* 27, 596-600. 10.1080/15257770802136065 18600511

[DMM029488C58] Takiue Y., Hosoyamada M., Yokoo T., Kimura M. and Shibasaki T. (2008b). Progressive accumulation of intrinsic mouse uromodulin in the kidneys of transgenic mice harboring the mutant human uromodulin gene. *Biol. Pharm. Bull.* 31, 405-411. 10.1248/bpb.31.405 18310901

[DMM029488C59] Trudu M., Janas S., Lanzani C., Debaix H., Schaeffer C., Ikehata M., Citterio L., Demaretz S., Trevisani F., Ristagno G. et al. (2013). Common noncoding UMOD gene variants induce salt-sensitive hypertension and kidney damage by increasing uromodulin expression. *Nat. Med.* 19, 1655-1660. 10.1038/nm.3384 24185693 PMC3856354

[DMM029488C60] Tsang K. Y., Chan D., Bateman J. F. and Cheah K. S. E. (2010). In vivo cellular adaptation to ER stress: survival strategies with double-edged consequences. *J. Cell Sci.* 123, 2145-2154. 10.1242/jcs.068833 20554893

[DMM029488C61] Turner J. J. O., Stacey J. M., Harding B., Kotanko P., Lhotta K., Puig J. G., Roberts I., Torres R. J. and Thakker R. V. (2003). UROMODULIN mutations cause familial juvenile hyperuricemic nephropathy. *J. Clin. Endocrinol. Metab.* 88, 1398-1401. 10.1210/jc.2002-021973 12629136

[DMM029488C62] Vylet'al P., Kublová M., Kalbáčová M., Hodaňová K., Barešová V., Stibůrková B., Sikora J., Hůlková H., Živný J., Majewski J. et al. (2006). Alterations of uromodulin biology: a common denominator of the genetically heterogeneous FJHN/MCKD syndrome. *Kidney Int.* 70, 1155-1169. 10.1038/sj.ki.5001728 16883323

[DMM029488C63] Weinman E. J., Eknoyan G. and Suki W. N. (1975). The influence of the extracellular fluid volume on the tubular reabsorption of uric acid. *J. Clin. Invest.* 55, 283-291. 10.1172/JCI107931 1127100 PMC301746

[DMM029488C64] Williams S. E., Reed A. A. C., Galvanovskis J., Antignac C., Goodship T., Karet F. E., Kotanko P., Lhotta K., Moriniere V., Williams P. et al. (2009). Uromodulin mutations causing familial juvenile hyperuricaemic nephropathy lead to protein maturation defects and retention in the endoplasmic reticulum. *Hum. Mol. Genet.* 18, 2963-2974. 10.1093/hmg/ddp235 19465746 PMC2714724

[DMM029488C65] Wolf M. T. F., Wu X.-R. and Huang C.-L. (2013). Uromodulin upregulates TRPV5 by impairing caveolin-mediated endocytosis. *Kidney Int.* 84, 130-137. 10.1038/ki.2013.63 23466996 PMC3700562

[DMM029488C66] Wu X. W., Muzny D. M., Lee C. C. and Caskey C. T. (1992). Two independent mutational events in the loss of urate oxidase during hominoid evolution. *J. Mol. Evol.* 34, 78-84. 10.1007/BF00163854 1556746

